# The Multi-Partner Consortium to Expand Dementia Research in Latin America (ReDLat): Driving Multicentric Research and Implementation Science

**DOI:** 10.3389/fneur.2021.631722

**Published:** 2021-03-11

**Authors:** Agustin Ibanez, Jennifer S. Yokoyama, Katherine L. Possin, Diana Matallana, Francisco Lopera, Ricardo Nitrini, Leonel T. Takada, Nilton Custodio, Ana Luisa Sosa Ortiz, José Alberto Avila-Funes, Maria Isabel Behrens, Andrea Slachevsky, Richard M. Myers, J. Nicholas Cochran, Luis Ignacio Brusco, Martin A. Bruno, Sonia M. D. Brucki, Stefanie Danielle Pina-Escudero, Maira Okada de Oliveira, Patricio Donnelly Kehoe, Adolfo M. Garcia, Juan Felipe Cardona, Hernando Santamaria-Garcia, Sebastian Moguilner, Claudia Duran-Aniotz, Enzo Tagliazucchi, Marcelo Maito, Erika Mariana Longoria Ibarrola, Maritza Pintado-Caipa, Maria Eugenia Godoy, Vera Bakman, Shireen Javandel, Kenneth S. Kosik, Victor Valcour, Bruce L. Miller

**Affiliations:** ^1^The Global Brain Health Institute (GBHI), University of California, San Francisco, San Francisco, CA, United States; ^2^The Global Brain Health Institute (GBHI), Trinity College Dublin, Dublin, Ireland; ^3^Cognitive Neuroscience Center, Universidad de San Andrés, Buenos Aires, Argentina; ^4^National Scientific and Technical Research Council (CONICET), Buenos Aires, Argentina; ^5^School of Psychology, Center for Social and Cognitive Neuroscience, Latin American Institute for Brain Health (BrainLat), Universidad Adolfo Ibanez, Adolfo Ibanez University, Santiago, Chile; ^6^Department of Neurology, Memory and Aging Center, University of California, San Francisco, San Francisco, CA, United States; ^7^Department of Radiology and Biomedical Imaging, University of California, San Francisco, San Francisco, CA, United States; ^8^Psychiatry Department, School of Medicine, Aging Institute, Pontificia Universidad Javeriana, Bogotá, Colombia; ^9^Memory and Cognition Clinic, Intellectus, Hospital Universitario San Ignacio, Bogotá, Colombia; ^10^Mental Health Unit, Hospital Universitario Santa Fe de Bogotá, Bogotá, Colombia; ^11^Grupo de Neurociencias de Antioquia, Universidad de Antioquia, Medellín, Colombia; ^12^Cognitive and Behavioral Neurology Unit, Hospital das Clinicas, University of São Paulo Medical School, São Paulo, Brazil; ^13^Unit Cognitive Impairment and Dementia Prevention, Cognitive Neurology Center, Peruvian Institute of Neurosciences, Lima, Perú; ^14^Instituto Nacional de Neurologia y Neurocirugia MVS, Universidad Nacional Autonoma de Mexico, Mexico, Mexico; ^15^Department of Geriatrics, Instituto Nacional de Ciencias Médicas y Nutrición Salvador Zubirán, Mexico, Mexico; ^16^Univ. Bordeaux, Inserm, Bordeaux Population Health Research Center, Bordeaux, France; ^17^Centro de Investigación Clínica Avanzada, Hospital Clínico, Facultad de Medicina Universidad de Chile, Santiago, Chile; ^18^Departamento de Neurología y Neurocirugía, Hospital Clínico Universidad de Chile, Santiago, Chile; ^19^Departamento de Neurociencia, Facultad de Medicina Universidad de Chile, Santiago, Chile; ^20^Clínica Alemana Santiago, Universidad del Desarrollo, Santiago, Chile; ^21^Geroscience Center for Brain Health and Metabolism (GERO), Santiago, Chile; ^22^Neuropsychology and Clinical Neuroscience Laboratory, Physiopathology Department, Institute of Biomedical Sciences, Neuroscience and East Neuroscience, Santiago, Chile; ^23^Faculty of Medicine, University of Chile, Santiago, Chile; ^24^Memory and Neuropsychiatric Clinic (CMYN) Neurology Department, Faculty of Medicine, Hospital del Salvador, University of Chile, Santiago, Chile; ^25^Hudson Alpha Institute for Biotechnology, Huntsville, AL, United States; ^26^Facultad de Medicina, Universidad de Buenos Aires, Buenos Aires, Argentina; ^27^ALZAR – Alzheimer, Buenos Aires, Argentina; ^28^Facultad Ciencias Médicas, Instituto Ciencias Biomédicas, Universidad Católica de Cuyo, San Juan, Argentina; ^29^Hospital Santa Marcelina, São Paulo, São Paulo, Brazil; ^30^Multimedia Signal Processing Group - Neuroimage Division, French-Argentine International Center for Information and Systems Sciences, Rosario, Argentina; ^31^Faculty of Education, National University of Cuyo, Mendoza, Argentina; ^32^Universidad del Valle, Cali, Colombia; ^33^Ph.D. Program in Neuroscience, Department of Psychiatry, Physiology, Pontificia Universidad Javeriana, Bogotá, Colombia; ^34^Departamento de Física, Facultad de Ciencias Exactas y Naturales, Universidad de Buenos Aires, Buenos Aires, Argentina; ^35^Department of Molecular, Cellular, and Developmental Biology, Neuroscience Research Institute, University of California, Santa Barbara, Santa Barbara, CA, United States

**Keywords:** dementia, fronto-temporal dementia, SES, SDOH, genetics, Alzheimer's disease, implementation science, Latin America

## Abstract

Dementia is becoming increasingly prevalent in Latin America, contrasting with stable or declining rates in North America and Europe. This scenario places unprecedented clinical, social, and economic burden upon patients, families, and health systems. The challenges prove particularly pressing for conditions with highly specific diagnostic and management demands, such as frontotemporal dementia. Here we introduce a research and networking initiative designed to tackle these ensuing hurdles, the Multi-partner consortium to expand dementia research in Latin America (ReDLat). First, we present ReDLat's regional research framework, aimed at identifying the unique genetic, social, and economic factors driving the presentation of frontotemporal dementia and Alzheimer's disease in Latin America relative to the US. We describe ongoing ReDLat studies in various fields and ongoing research extensions. Then, we introduce actions coordinated by ReDLat and the Latin America and Caribbean Consortium on Dementia (LAC-CD) to develop culturally appropriate diagnostic tools, regional visibility and capacity building, diplomatic coordination in local priority areas, and a knowledge-to-action framework toward a regional action plan. Together, these research and networking initiatives will help to establish strong cross-national bonds, support the implementation of regional dementia plans, enhance health systems' infrastructure, and increase translational research collaborations across the continent.

## Dementia Research in Latin America: Toward Unraveling the Unique Genetic and Environmental Factors

The prevalence and incidence of dementia appears to be stable or declining in the US and other high income countries (HIC) (1–3), where cohorts being studied typically consist of relatively homogeneous populations with middle/high social determinants of health (SDH), including socioeconomic status (SES) (4, 5). Latin American countries (LAC) are marked by an opposite scenario (2, 3, 6–10), with increased dementia prevalence amidst a fast demographic shift (3, 11). Together, residents of Argentina, Brazil, Chile, Colombia, Mexico, and Peru make up more than 75% of the region's population. This rise may be driven by unique genetic factors and unfavorable SDH in the region which may influence the prevalence and presentation of dementia (4, 9, 11–16). Across the region, the case of frontotemporal dementia (FTD) is even more challenging than Alzheimer's dementia (AD).

Environmental factors seem to be critical for dementia presentation in the region. SDH may selectively impact dementia in LAC (11, 12) by modulating cognitive progression and brain health burden. However, available reports on SDH have not used sophisticated cognitive and imaging measures, and scant evidence comes from LAC (2, 11). The region presents an important opportunity to study these questions because of the greater disparities in SES and SDH compared to HIC (2, 11). To address these pressing needs without overlooking the region's heterogeneity, harmonized data must be collected from several countries with different SDH levels. This poses a unique challenge for clinical characterization, as these factors will strongly influence dementia presentation (17). Traditional markers of disease severity, including informant ratings, cognitive performance (executive, memory, and social cognition), and neuroimaging features, should be interpreted in the context of SDH factors. Our consortium has developed core composite measures of SDH capturing the heterogeneity of different factors, including *SES* (food & housing insecurity, access to foods that support health eating habits), *education* (Early childhood development, language and literacy, higher education), *health and health care* (access to health services, health literacy), occupation (lifetime employment history, employment status), and *social and community context* (discrimination, social cohesion, crime and violence).

At the same time, genetic factors also seem to drive dementia presentation in LAC, with apparent stronger familial aggregation of dementia compared with HIC. The region hosts some of the largest populations of familial dementing disorders, and some populations may harbor unique genetic influences conferring increased risk of dementia (4, 9, 11, 15, 18–20). Long isolation periods, endogamy, and the admixture of different ancient populations provide unique opportunity to assess genetic-environmental influences in heterogeneous samples (11, 19). Genetic studies in Latin-American immigrants have shown large effect sizes (14) but these studies have not been explored at a regional level (21). Large consortia have assessed genetic susceptibility mostly in HIC, but other regions, including LAC (4, 11) still remain understudied. The recent development of polygenic risk scores (PRS) to identify individuals at risk for dementia in developed countries are very promising, but they lack validation in more heterogeneous samples (22–25). Our group has found multiple genetic influences of dementia (16, 19, 26–53). The identification of new families may have a long-term impact on therapeutic initiatives (19). Assessing genetic markers, combining common and novel variants, as well as future development of PRS in LAC populations, will bring valuable knowledge about neurogenetic determinants of dementia.

The ways in which the combination of genetic and SDH-related risks interact in the dementia presentation across LAC is not well-understood. New studies in this region are needed to identify novel genetic and gene-environment interactions (i.e., genetic interactions with LAC-specific geography and SDH) leading to dementia, and novel pathways applicable to regional therapies. LAC face a dearth of innovative, harmonized, and cross-regional studies on AD and FTD, and establishing multi-center LAC initiatives is critical for global discovery and research harmonization in these underrepresented populations. Yet, region-specific determinants remain uncharted and, due to different factors (54, 55) the region is still underrepresented in international publications (11). Thus, given that dementia research critically calls for a more global perspective (4), there is an urgent need to compare US and LAC samples via integrative approaches. The development of a more extended regional network, based on multi-institutional harmonized research (11), is crucial for the field.

## The ReDLat Approach

**Figure 1:**
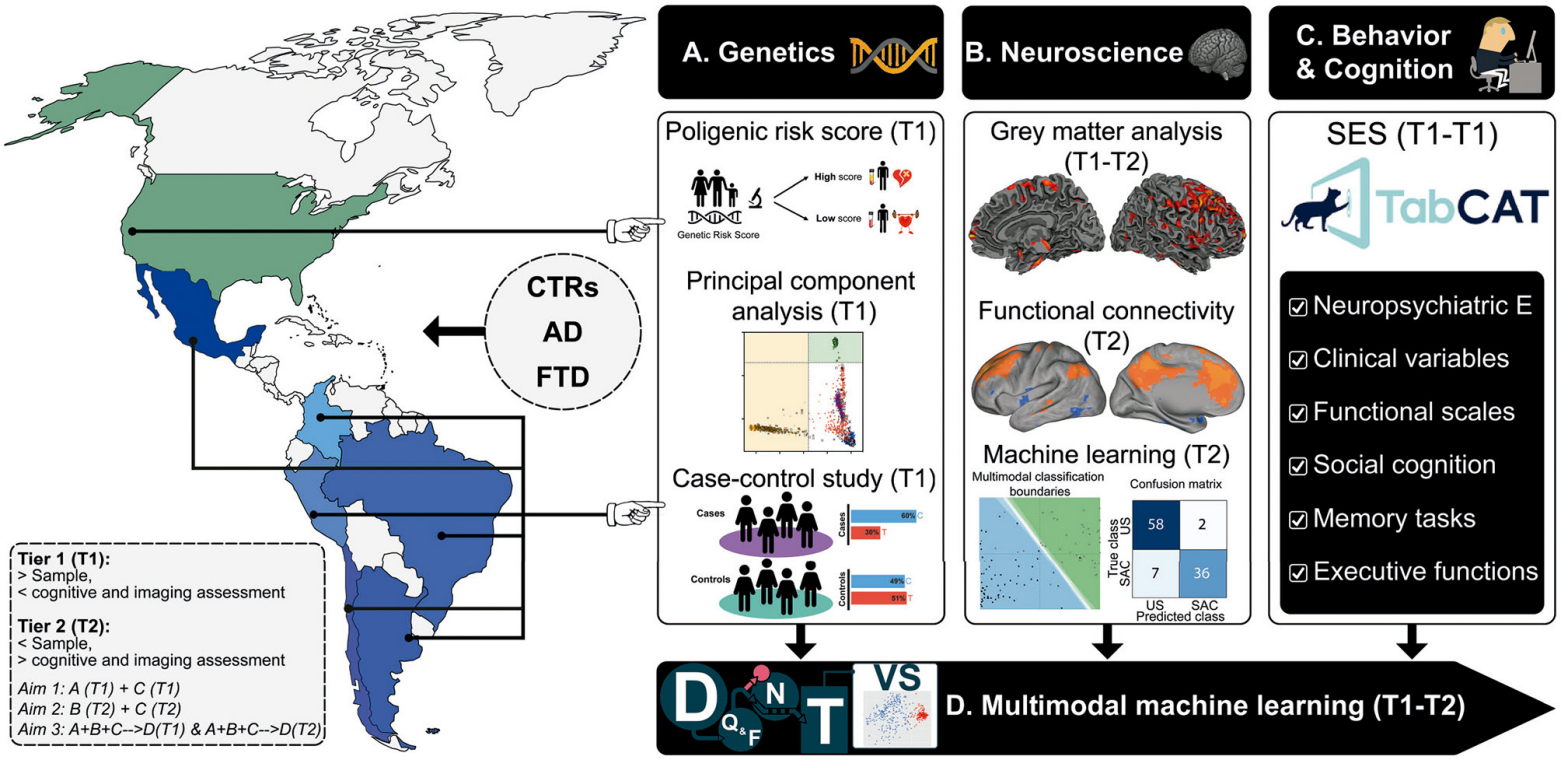
The ReDLat initiative. Systematic comparisons between LAC and US samples of AD and FTD via a novel, multimodal approach. The multimodal patterns will be assessed with different measures of **(A)** genetic risk (Aim 1), **(B)** imaging markers boosted by computational approaches, and **(C)** harmonized and novel measures of cognitive profiles and SDH (Aim 2). These data sources will be **(D)** integrated and compared among countries through machine learning (Aim 3) to unveil the main commonalities and differences between US and LAC samples. Tier 1 (T1): Larger study (Aim 1 & 3). Tier 2 (T2, smaller study with deep neurocognitive investigation (Aim 2 & 3). D, Data; Q&F, Quality & feature extraction; N, normalization; T, test; VS, visualization; Neuropsychiatric E, Neuropsychiatric evaluations. Reproduced with authorization from (1).

The Latin America and the Caribbean Consortium on Dementia [LAC-CD (1)], in association with world-class researchers from the US has developed an agenda to tackle the unique genetic and SDH risk for dementia in LAC (2). In response to this call, the Multi-partner consortium to expand dementia research in Latin America (ReDLat, supported by the NIH-NIA, the Alzheimer's Association, the Rainwater Charitable Foundation, and Global Brain Health Institute) is aimed to identify the unique genetic and SDH factors that drive AD and FTD presentation in LAC relative to the US, including risk factors, cognitive profiles and brain imaging (Figure 1). To this end, we are establishing a first-in-class cohort anchored in six LAC (Argentina, Chile, Colombia, Brazil, Mexico, and Peru), compared to US samples (totaling > 4,200 participants, including 2,100 controls, 1,050 AD patients, and 1,050 FTD patients), led by world-renowned leaders in dementia research. We couple standardized clinical assessments with innovative analytical techniques to account for heterogeneity in these diverse populations. By combining standardized genetic, neuroimaging, and behavioral (cognitive and SDH) measures, we will investigate whether there are unique risk factors for AD and FTD in LAC (e.g., genetic risk factors enriched in LAC populations; underlying cognitive and neural vulnerability due to SDH) compared to US populations. Our plan to recruit large numbers of controls and patients across these diverse populations will provide excellent opportunities to identify new genetic and SDH risks for AD and FTD. In addition, the machine learning strategies we are developing to reduce the impact of background heterogeneity will allow us to refine the accuracy of our association studies.

Our first specific aim is to establish genetic contributions to AD and FTD in diverse LAC cohorts. First, by elucidating the genetic substructure and familial contributions to AD and FTD in LAC relative to the US, we will be able to identify proper populations for replication of our genetic findings. We anticipate that, relative to the US, LAC will have a higher frequency of familial forms of AD and FTD. Discovery of new families with multiple affected individuals will advance efforts to treat AD and FTD in patients with rare mutations. Second, by assembling this large cohort, we will also be well-positioned to establish a preliminary LAC-specific polygenic risk score (PRS) for predicting AD and FTD risk in future samples. We expect that PRS will work best at discriminating patients from controls in the European predominant subpopulation (US and, to a lesser extent, Argentina, Chile) than in the African and Indigenous-majority admixed cohorts (Peru, Brazil, Colombia, Mexico).

In our second specific aim, we will elucidate the impact of SDH on clinical, cognitive, and brain imaging signatures in LAC and the US. To compare patients across regions, we establish standardized neurocognitive measures and harmonization protocols to understand how SDH impacts the manifestations of dementia in LAC. First, we will evaluate how SDH moderates the relationship between age at onset and disease severity in AD and FTD. We anticipate that AD and FTD will emerge at an earlier age in low-SDH vs. high-SDH (dichotomized) patients, and measures of disease severity, including cognitive performance, and multimodal neuroimaging, will be worse in the low-SDH group even after accounting for age. We expect that difference in disease severity ratings, cognition, and multimodal neuroimaging that reflect low vs. high SDH disparities will be greater in LAC patients compared to US patients. Latin America constitute the region with the largest inequalities in the world (56). Moreover, SES/SDH represent a strong influence on dementia risk (2, 57).

**Figure 2:**
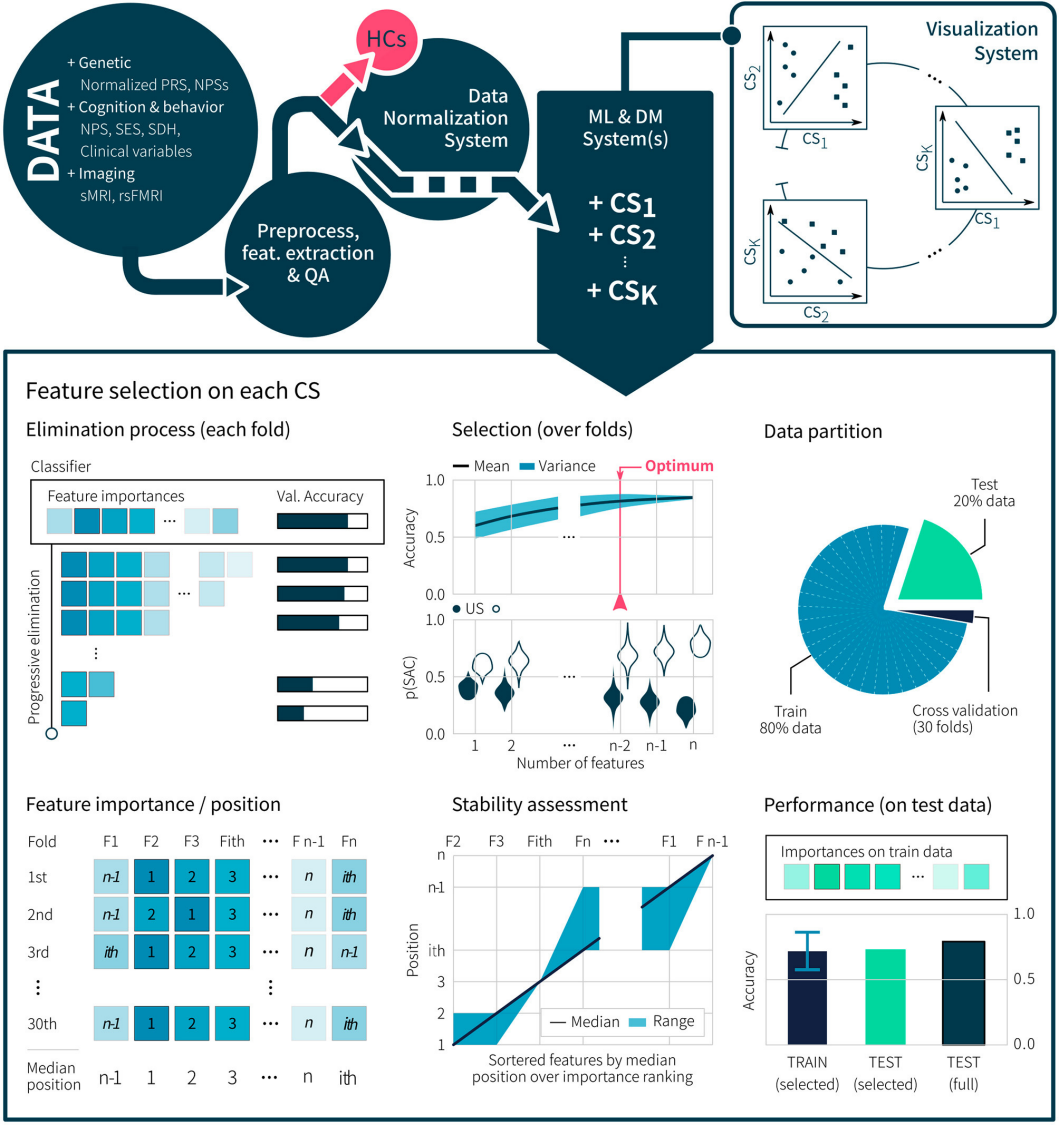
Machine Learning approach for the discovery of discriminant multidomain features between US and LAC patients with AD and FTD. Genetic, cognitive, SES, SDH, and imaging data are preprocessed with specific normalization methods to extract reliable features. After a feature-based quality assessment (QA), samples are separated in healthy controls (HCs) and patients. HC samples are used to apply normalization and harmonization methods over patient samples, enabling the correction of site-dependent bias in the data. Then, machine learning (ML) and data mining (DM) methods are used for multi-domain classification systems (CS), to find robust features, and to develop visualization dashboards. Each CS performs a progressive feature elimination process to find the most important features and to assess the stability of the model performance using a k-fold cross-validation over the training partition. Finally, performance and generalization in the classification are assessed via test independent partition from the training set.

Our last specific aim seeks to determine whether genetic risk and SDH yield better discrimination between LAC and US patients as compared with other cognitive, neuroimaging, and clinical variables. To our knowledge, no study has sought to establish which potential predictors prove more sensitive to discriminate between LAC and US patients. In particular, although genetic risk and SDH have the potential to robustly differentiate between such samples, no study has explored their combined role, let alone as compared to other multimodal factors. To address this issue, we will apply data-driven machine-learning pipelines to determine top factors that best discriminate patients in LAC from those in the US (Figure 2). Multimodal measures from controls of each country will be used for population-specific normalization of patient data. We anticipate that the top features, better discriminating LAC from US patients will be related to SDH and genetic risk (e.g., standardized PRS) in comparison to other variables (clinical, cognitive, and imaging measures).

ReDLat will establish a large LAC cohort of harmonized, well-characterized AD and FTD patients and controls. We anticipate the development of a better understanding of genetic and environmental contributions to neurocognitive manifestations of dementia and the identification of novel targets for risk reduction and disease prevention in LAC. Our large multimodal, cross-sectional study will enable clinical assessment of understudied patient groups, extend and harmonize existing data sets, prompt the development of novel measures, and inform future work on the clinical value of combined multimodal profiles to predict disease presentation and progression in longitudinal studies of diverse populations.

## ReDLat Ongoing Progress and Extensions

**Figure 3:**
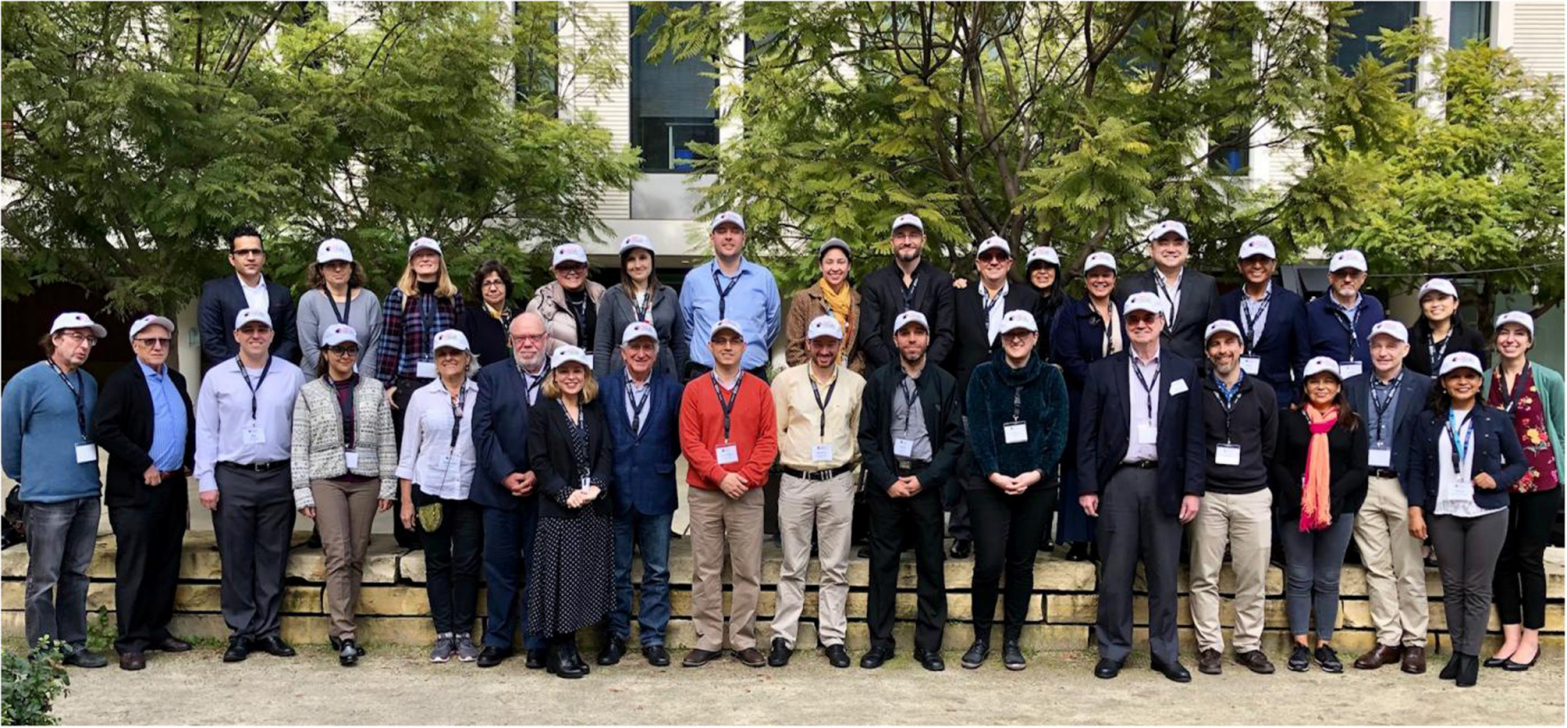
ReDLat Kickoff meeting at San Francisco, CA. On January 27 2020, regional leaders, local investigators, ReDLat members, as well as authorities from the Global Brain Health Institute (GBHI), Alzheimer's Association, the Tau Consortium, the National Institute of Health (NIH) and other organizations met at UCSF in a US-Latin American Networking on Dementia Symposium. Co-hosted by GBHI and the UCSF Memory and Aging Center, the symposium served to launch ReDLat. Reproduced with authorization from (58).

On January 27, 2020, a kickoff meeting (Figure 3) involved more than 50 leaders in dementia from Latin America and community members from the Global Brain Health Institute (GBHI), Alzheimer's Association, the Tau Consortium, the National Institute of Health (NIH) and private companies at UCSF Mission Bay (58). Since then, ReDLat has been led by an Executive Committee (EC), with working groups (biospecimen handling, cognitive & clinical assessments, neuroimaging, data management, research & publications, and finance), made up of representatives from each site, who meet bi-weekly to review progress, build consensus and address issues as they arise. The ReDLat taskforce (EC and working groups involving more than 90 people) guarantees a shared decision-making process and equal distribution of opportunities for involved centers.

Substantial effort has been devoted to developing strategies for harmonization of participant enrollment across sites. We created a detailed study-wide protocol (see Supplementary Data 1) and site-specific manuals of operation to ensure accurate and consistent collection. In addition to outlining recruitment procedures and requirements for personnel, the manual provides step-by-step instructions for completing each assessment, processing and shipping specimens, and collecting harmonized neuroimaging data. We adapted the standardized diagnostic assessment used at the UCSF Memory and Aging Center to align with the local sites' procedures (see Supplementary Data 2). The instrument is brief enough to be completed in full for every enrolled participant and will incorporate impressions from the physician who examined the participant, with input from the evaluating neuropsychologist. We also hold in-depth training for personnel at each site, covering neuropsychological testing, clinical assessments, DNA extraction, image acquisition, and genealogy collection procedures, among others. Time is set aside to ensure access to all technological platforms and to teach staff about accurate and timely entry of data into the central database. Videos detailing these instructions are available to each site for ongoing training purposes. Site staff who complete this training are certified to assess participants and this certification will be renewed on an annual basis, either in-person or via video recording, to minimize drift over time. While these trainings were conducted in person for several sites, travel restrictions due to COVID-19 required us to transition to a virtual format.

We have worked with the National Alzheimer's Coordinating Center to obtain permission for adaptation of the Uniform Data Set Cognitive Assessment to Spanish and Portuguese. Based on feedback from investigators, we adapted the language for some tasks to optimize cultural appropriateness at each site. We developed a new instrument to systematically assess SES and SDH. This UCSF-ReDLat questionnaire has been culturally adapted with input from each enrolling site, based on previous reports as well as national censuses from Colombia, Brazil, Chile, and Perú and following cross-cultural implementation recommendations (59–64). The questionnaire captures educational attainment, race and ethnicity, health literacy, financial strain, food insecurity, housing insecurity, childhood trauma, social connections, social isolation, access to healthcare, occupation, and employment status.

**Figure 4:**
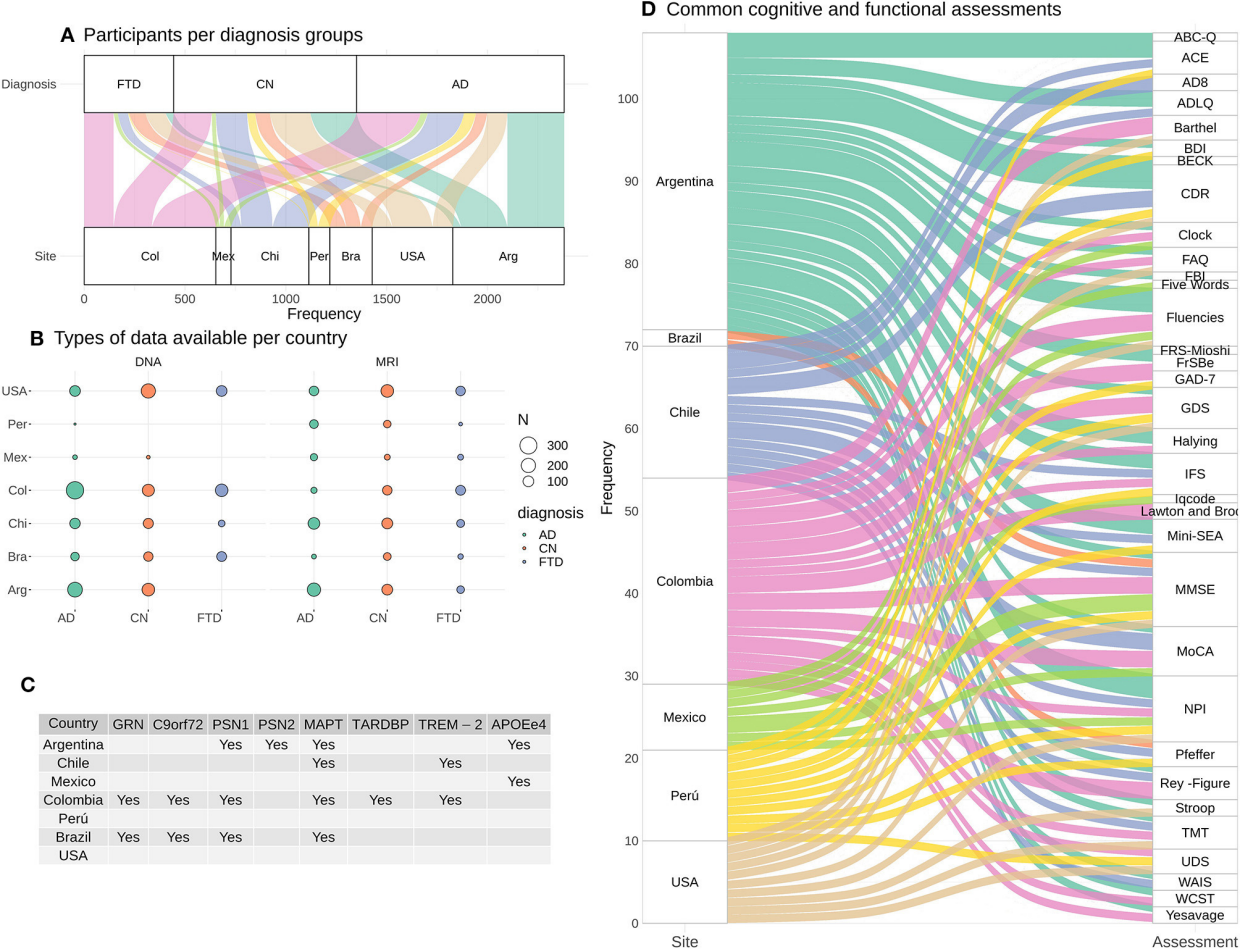
ReDLat pre-existing data. **(A)** Estimates of cases with MRI (T1, rs-fMRI, or DTI) and/or DNA per country. **(B)** Number of participants with DNA and MRI data per diagnosis and per country. **(C)** Mutations already identified across countries. **(D)** Summary of the cognitive and functional assessments available in each country.

Based on the data sharing process detailed above, we performed preliminary analysis. With respect to genetics, we have identified multiple new families with different mutations including *PSEN1, PSEN2, TARDBP, GRN, TREM2, MAPT, EPO4*, and C9orf72 (see Figure 4C). Regarding the use of machine learning for combination of neuroimaging modalities as well as behavioral/cognitive assessment, we have developed several pipelines with preliminary data and other samples (65–71). We plan to develop a semi-empirical whole-brain multimodal computational approach (MRI, DTI, and fMRI) with mathematical modeling for characterization of global brain dynamics restricted by structural priors (72). This model will also allow data augmentation (73, 74) amplifying the expansion of our machine learning protocol. Regarding SDH and cognitive assessment, we have shown the power of social cognition and SDH (64) in predicting brain health. We have also developed complementary measures of emotion processing (75), and preliminary assessments of naturalistic speech (70), and multi-country validation of our social cognition measures (76) for future assessment of our patients.

The assessment of affordable measures such as high-density EEG has emerged as a highly promising transdiagnostic and disease-specific approach for dementia (77–79). EEG provides highly affordable, non-fatiguing, non-invasive measures which can reveal early deficits traceable to well-established neurophysiological processes affected across conditions. Our taskforce has developed expertise in EEG markers, including ERPs, oscillations, connectivity measures, source space analysis, decoding and machine learning approaches, for both active tasks and resting state recordings (29, 35, 80–117) alone or in combination with other technics (35, 81, 82, 87, 89, 89, 112, 118–120). In a regional project based on the ReDLat platform and additionally supported by Takeda, we will extend the protocol to compare multimodal EEG markers. We will explore the robustness of such markers (in comparisons with cognitive and neuroimaging markers) to discriminate between patients (AD and FTD) as well as disease severity and familial status. Also, using multi-feature machine learning, we will combine the ReDLat approaches (using neuroimaging) with EEG features to predict disease subtype, status and severity.

Given the current challenges triggered by the global pandemic, our group has taken advantage of this time to identify new opportunities for expanding the platform use by integrating existing datasets with genetic, cognitive, and imaging analyses of samples in hand. Figure shows an estimate of cases with MRI (4A: T1, rs-fMRI, or DTI) and/or DNA per country. All countries have data from participants that belong to AD, FTD and healthy controls. Figure 4B shows the number of participants with DNA and MRI data per diagnosis and per country. In total, an estimate of 2,208 participants have DNA data, and 1,349 participants have MRI data across diagnosis groups and across countries. Figure 4C provides a summary of the mutations that have already been found in patients and/or patient relatives. Figure 4D highlights a summary of the cognitive and functional assessments used in each country. These preexisting datasets will guarantee the continuity of ReDLat research during total or partial lockdowns.

## LAC-CD: Toward Networking, Implementation Science, and Capacity Building

RedLat is also aimed to develop implementation science and capacity building, and several actions has been performed via regional networking, training, and development of educational projects. This is the main goal of the Latin America and the Caribbean Consortium on Dementia [LAC-CD (1)], the regional organization where ReDLat was built. LAC-CD focuses on (a) training health practitioners in dementia field, (b) establishing networks to support multicentric research and clinical practice, (c) harmonizing clinical approaches to diagnosis and post-diagnostic support, (d) validating these approaches in unique populations, (e) increasing the appeal of regional and international grant proposals emerging from LAC networks rather than from individual groups, (f) accelerating access to knowledge and evidence-based decisions via a unified platform, (g) setting up effective communication channels to persuade heads of governments and private agencies of the need for integration and support via national and regional dementia strategies.

Nowadays, the consortium is promoted by the Alzheimer's Association and the Global Brain Health Institute, and holds more than 240 regional members. LAC-CD involves national representatives working in specific priority areas including dementia biomarkers, genetics and epidemiology, a dementia data platform, a clinical trial program, non-pharmacological interventions, and translational research networks. LAC-CD initiatives include (a) empowering local groups, (b) boosting coordinated efforts across the region, and (c) developing a Knowledge-to-Action Framework to develop a regional action plan.

### Empowering Local Groups: Education, Visibility, and Capacity Building

A key ambition of our consortium is to create harmonized approaches to dementia diagnosis in order to allow multi-country comparisons. First, we developed diagnostic recommendations (relevant clinical, neuropsychological, and behavioral assessments), for diagnosis of AD and FTD across LAC even where there are no available resources required for classification of dementias (121). Then, supported by the Inter-American Developmental Bank (IDB) and a GBHI pilot funding, we develop a best practice manual for dementia diagnosis^1^. The manual has been highlighted by the Alzheimer's & Dementia journal (122) and involves more than 40 leaders from expert panels and authors. The manual provides a regional approach to dementia in the region, its epidemiology and different health systems, clinical and neuropsychological assessments and a chapter on carers.

Several initiatives are being created to expand the visibility and dissemination of the consortium activities, including a LAC-CD website (http://lac-cd.org/, in English, Spanish, and Portuguese) providing information on projects, membership, news, opportunities, dissemination products, press releases, and social media. With the Alzheimer's Association, we have launched a LAC-CD – ISTAART webinar including an annual meeting, and periodic webinars focused on different topics.

Regarding capacity building, a Latin American Institute for Brain Health (BrainLat^2^) will be launched in Chile in 2021 by the University Adolfo Ibanez (UAI). BrainLat will bring together leading national and international institutions to develop support for the ReDLat and LAC-CD expansion and to develop world-class research in brain health. BrainLat will support the Latin American research on dementia with annual seed projects, postdoc positions, infrastructure support, a PhD program, neuroscientific equipment, and permanent support for 16 full research positions.

### Boosting Brain Health Coordinated Efforts Across the Region

**Figure 5:**
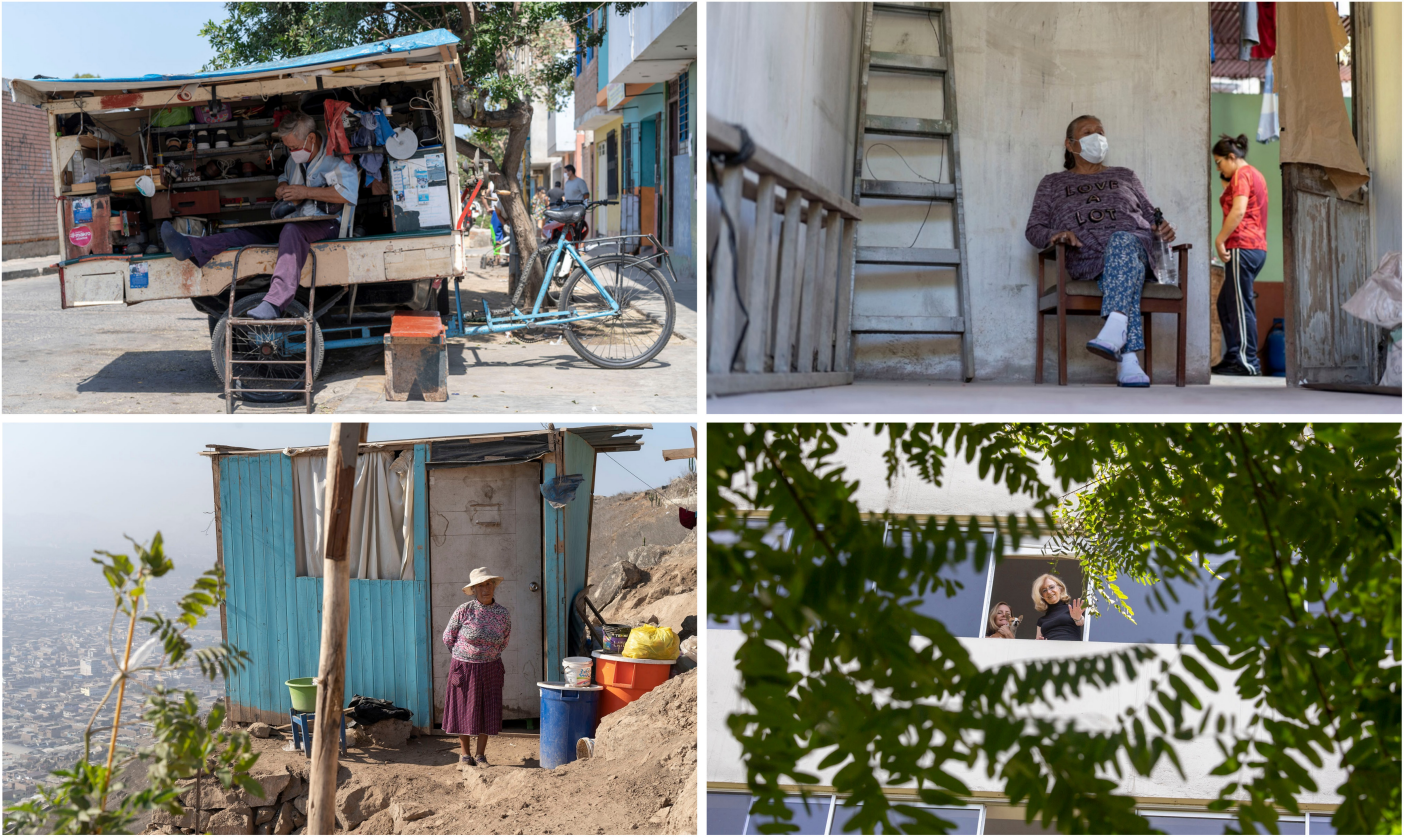
Testimonies from Peru highlighting different dimensions of the coronavirus outbreak and their impact on older people, and patients with cognitive decline and their families. The pictures above illustrate the people's vulnerabilities and the unpreparedness of the health system. Top left inset: Enrique (64 years old, Trujillo) suffers from diabetes mellitus but has been unable to get medication for 2 months. He is a shoe repairer with a small mobile stall and, after months of quarantine, he has to go out to work. Top right inset: Juana (64 years old, Trujillo) is a merchant diagnosed with coronavirus 3 months ago, which led to her needing supplemental oxygen and intravenous medications. Given the collapse of the hospitals, she was treated at home by her daughter. She thought she might lose her life, unable to perform simple activities (such as walking and eating) without great effort. Bottom left inset: Enedina (65 years old, Lima) lives with her youngest son who lost his job due to the pandemic restrictions. They live in a precarious room, without electricity, water or drainage. Bottom right Inset: On the other side of Lima, 83-year-old Mrs. Rosita lives with her family in a wealthy district. Her daughter has noted typical dementia symptoms, which have exacerbated since the quarantine. She doesn't understand the isolation, needs constant monitoring and urgently requires a neurological evaluation, but there are no services available due to the pandemic. Photos and testimonies from Peru documented by Alexander Kornhuber and Maritza Pintado Caipa. Individuals and relatives portrayed in the photos have provided written consent for reproduction. Reproduced with authorization from (126).

Albeit the common dementia regional challenges, coordinated multilateral responses are scarce (2, 56). Coordinative efforts such as brain health diplomacy (BHD) and convergence science (123–125) can facilitate the integration of expertise, institutions and strategies between governments and non-governmental organizations (NGOs) in the region. An example of this is a call we develop to raise awareness of the long-term syndemic impact (Figure 5) of coronavirus in aging and dementia across LACs (56). Subsequently, we proposed specific coordinated actions LACs to reduce such impact and new upcoming challenges, including the development of inexpensive mass testing and actions related to telemedicine, care, and research (126).

**Figure 6:**
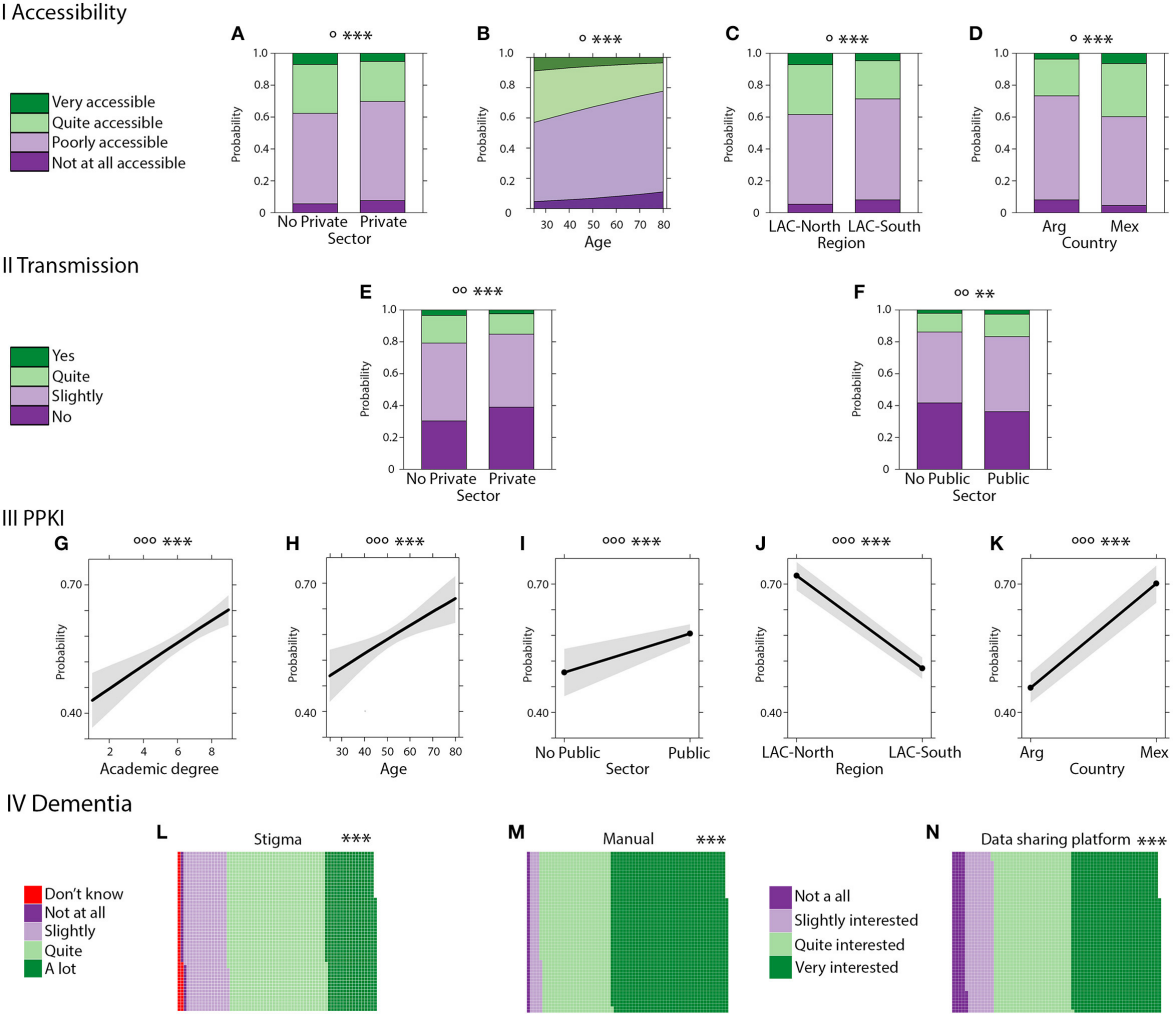
Dementia Public policies in Latin America. (I) Public policies accessibility. **(A)** Probability of response frequency regarding accessibility by sector. **(B)** Probability of response frequency regarding accessibility by age. **(C)** Probability of response frequency regarding accessibility by region. **(D)** Interaction of probability of response frequency of accessibility by country. (II) Public policies transmission. **(E)** Probability of response frequency regarding transmission by private sector. **(F)** Probability of response frequency regarding transmission by the public sector. (III) PPKI (public policy knowledge index). **(G)** Probability of response frequency regarding high PPKI by academic degree. **(H)** Probability of response frequency regarding high PPKI index by age. **(I)** Probability of response frequency regarding high PPKI by the public sector. **(J)** Probability of response frequency regarding PPKI by public region. **(K)** Probability of response frequency regarding PPKI by country. IV Aging. **(L)** Proportion of responses about aging stigma. **(M)** Proportion of responses about interest in aging and dementia manual. **(N)** Proportion of responses about interest in a data-sharing platform. Significance (*p* values): effects significance (**p ≤ 0.05, ***p ≤ 0.01), model significance (°*p* ≤ 0.1, °*p* ≤ 0.05, °*p* ≤ 0.01). Academic degree: 1: No reported education, 2: Technicians, 3: Tertiaries, 4; Certificates, 5: Undergrads, 6: Hospital Interns, 7: Post-graduate Specialization, 8: Master's Degree, 9: Ph.D. Reproduced with authorization from (127).

Another example of coordinated actions are related to surveying expert knowledge on dementia across different LACs. We recently assessed multiple dimensions of expert knowledge of health professionals working in aging across LACs (*N* = 3,365) and its modulation by different factors including expertise-related information (knowledge of public policies), individual differences (work, age, academic degree), and location across LACs (127). Results evidenced a tough knowledge gap of dementia at manifold levels (Figure 6) including lack of access and transmission of public health knowledge, stigma among professionals, and almost complete unawareness of innovative behavioral insights or nudges tools in public health domains. The survey also evidenced a critical need for regional manuals for best practices and data-sharing platforms for both clinical and research initiatives. These specific knowledge gaps and critical needs should be assessed by governmental agencies and NGOs to improve dementia knowledge at regional level.

### A Knowledge-to-Action Framework for a Regional Action Plan

**Figure 7:**
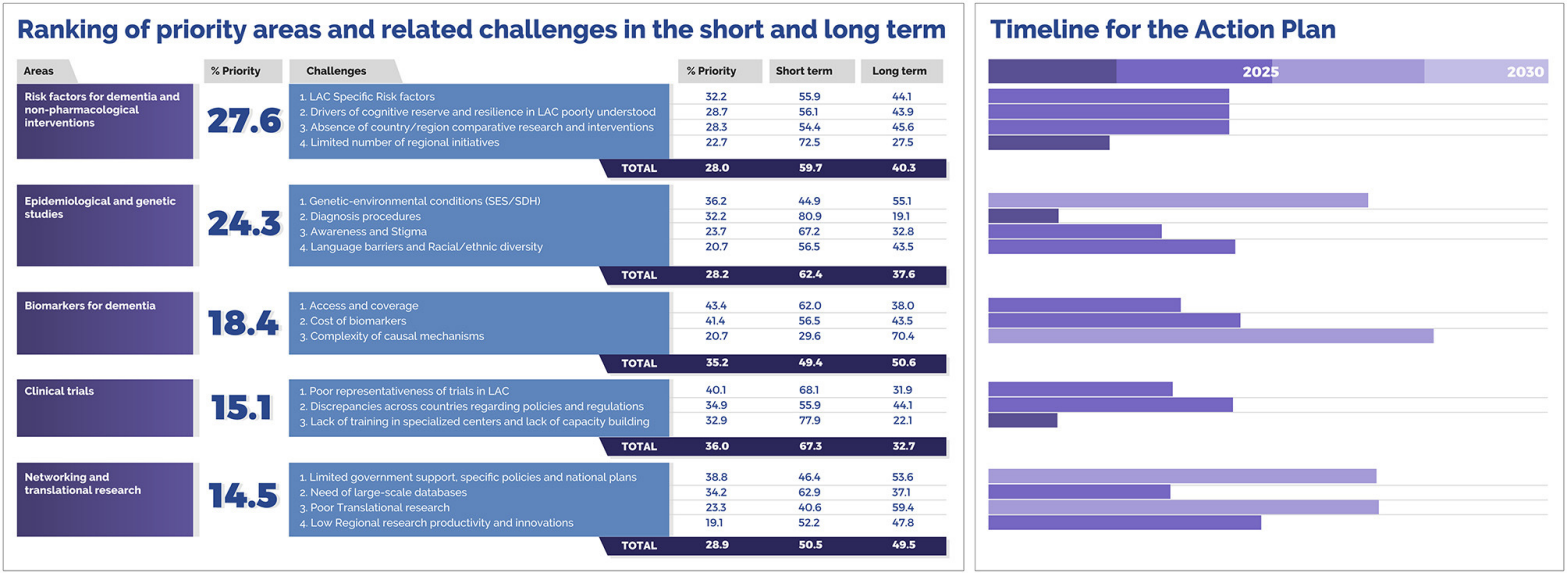
Priority levels assigned to core areas and challenges via a knowledge inquiry and related actions timelines. LAC-CD regional experts (*N* = 248) were presented with a survey and were asked to rank the 5 areas and associated challenges in order of priority. We calculated the percentage of respondents who rated these within the top two priorities and used these to rank both areas and challenges. The right inset shows the timeline for the proposed actions. Experts were also asked to deliver their views about a feasible timeline to address these challenges and actions (0–5 or 5–10 years) (% = Mean % of responses). Reproduced with authorization from (2).

LAC-CD has advanced a Knowledge to Action Framework (KtAF) toward regional action plan for dementia (2). Initially, we identified cross-regional priority areas (Figure 7), namely: (a) risk factors for dementia and non-pharmacological interventions, (b) epidemiological and genetic studies, (c) biomarkers for dementia, (d) clinical trials, and (f) networking and translational research. Evidence-based strategies were proposed to tackle ensuing challenges while considering key sources of complexity (genetic isolates, admixture in populations, environmental factors, and barriers to effective interventions). These strategies were mapped to the above priorities, laying the conceptual groundwork for our further KtAF. These procedures have been endorsed by experts as vehicles to third-generation knowledge (128).

**Figure 8:**
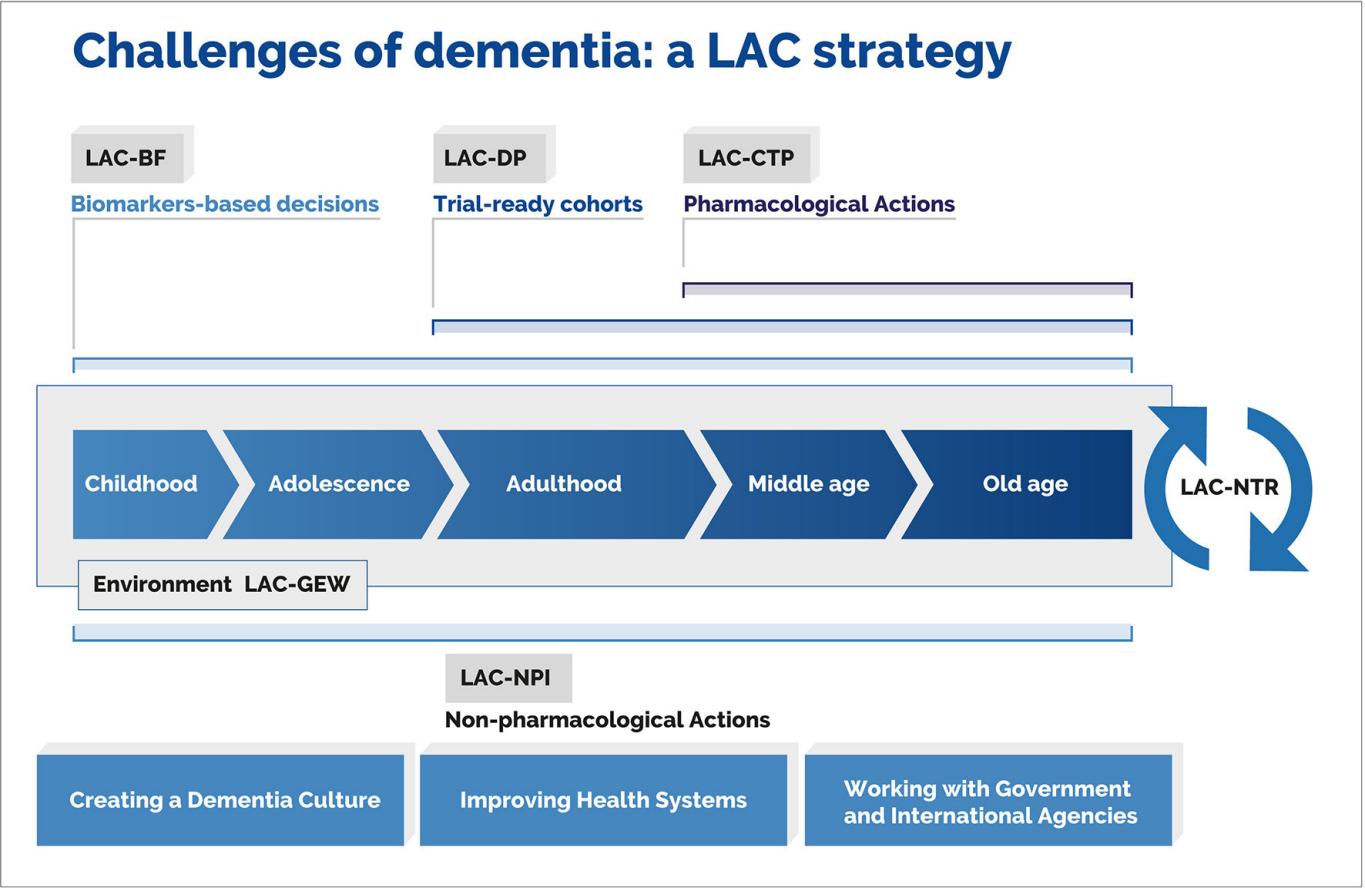
Knowledge-to-action framework. The diagram captures challenges posed by dementia and the related mapping of key actions. Such actions are linked to specific working groups that have been included in the framework. This approach comprises a biomarker framework (LAC-BF), genetics and epidemiology workgroup (LAC-GEW), dementia platform (LAC-DP), clinical trial program (LAC-CTP), non-pharmacological interventions (LAC-NPI), and an LAC network for translational research (LAC-NTR). Reproduced with authorization from (2).

The KtAF comprises five workgroups, each responsible for specific tasks (Figure 8). A Non-pharmacological Interventions Workgroup (LAC-NPI) will address regional risk factors. It will align with international initiatives, reinforce surveillance by incorporating the World Health Organization (WHO) STEPwise approach, foster national dementia plans across the region, develop research on cognitive reserve and resilience, and improve training and educational programs via the GBHI and the Alzheimer's Association. The Genetic and Epidemiology Workgroup (LAC-GEW) will aim to implement epidemiological studies across LACs, identify lifelong factors impacting neurocognitive development, expand family history and genetic protocols, develop a harmonized digital data-sharing platform, boost research on genetic heterogeneity, and support the creation of a regional LAC dementia observatory. The Biomarker Framework (LAC-BF) will strive to validate complementary affordable biomarkers against the A/T/N framework, focusing on cognitive assessment, eye-tracking, non-invasive peripheral markers (i.e., plasma markers.), and multimodal neuroimaging (e.g., EEG, MRI, fMRI, DTI) combined with machine- and deep-learning algorithms (65, 66, 68, 71, 82, 87, 112, 126, 129–134) and novel theoretical approaches (118, 119, 135–139). These unspecific markers can be validated with the measurement and comparison with A/T/N framework's canonical markers. Thus, affordable measures can support the validation of low-cost biomarkers in the region. The Clinical Trial Program (LAC-CTP) will identify main countries and hubs possessing the infrastructure for prevention trials, connect these programs with national regulatory agencies for regional harmonization, develop trial-ready cohorts across countries, and launch a clinical trial training program to empower less experienced centers. Finally, a Network for Translational Research (LAC-NTR) will promote translational research through a network of scientists, clinicians, pharmaceutical leaders, and government representatives. It will also develop digital platforms to maximize collaboration and exchange of resources, while promoting synergy among regional initiatives. Through the joint effort of these workgroups, the KtA will increase awareness, knowledge, and resources leading to global equity in the fight against dementia.

## Conclusions

Our recently launched consortium grasps relevant features for upcoming progress and expansion. Regarding *multidisciplinary innovation*, ReDLat focuses on the largely ignored convergence of genetic and SDH risks, especially considering multimodal (clinical, cognitive, neuroimaging) effects and innovative machine learning techniques. Regarding *translational impact*, our project is research-based, but geared to capacity building and implementation science (diagnosis, education, support, evidence-based policy), favoring regional commitment. We also aim to *empower local ideas in a global networking landscape*, as our initiative merges bottom-up LAC proposals into a single landscape. We promote win-win HIC-LMIC collaborations facing local needs from a local-global perspective. We also *focus on barriers*, by tackling HIC-LMIC cultural-communicative differences between researchers, assessing underrepresented populations, pushing changes regarding lack of trust among teams based on an equitable platform for collective decision making, and bringing collective support to minimize emergent leaders' disregards. We believe these actions promoting brain health and dementia across LACs and globally will help to truly transform challenges into opportunities.

## Author Contributions

AI designed the proposal under supervision of BM. AI wrote the drafts, discussed contributions from all co-authors. All authors searched the literature, participated in discussing the contents of the paper, contributed to editing, and approved the final version of the article.

## Conflict of Interest

The authors declare that the research was conducted in the absence of any commercial or financial relationships that could be construed as a potential conflict of interest.

## Footnotes

^1^The manual is available here: *http://lac-cd.org/en/2020/06/17/manual-for-best-practices-for-the-dementia-diagnosis/*

^2^https://brainlat.uai.cl
