# Supplementary material for: The Multi-Partner Consortium to Expand Dementia Research in Latin America (ReDLat): Driving Multicentric Research and Implementation Science
Source: Front Neurol. 2021 Mar 11;12:631722. doi: 10.3389/fneur.2021.631722 (PMC7992978; doi:10.3389/fneur.2021.631722)
Supplement: Supplementary file 1 [file Data_Sheet_1.PDF]

ReD-Lat

Multi-Partner Consortium to Expand Dementia Research in Latin America

Research Protocol

Version 1.1

Abbreviation List 3
A. PROTOCOL INFORMATION 3
Associate researchers, key personnel, OSC, and team structure: 5
Institutions and Reviewing IRB’s: 6
B. STUDY SUMMARY 6
General Aim and hypothesis 6
Specific Aims and Hypothesis 8
C. SUBJECTS AND INCLUSION/EXCLUSION CRITERIA 8
Participants with Dementia 9
Healthy Control Participants 10
Relatives 10
D. STUDY DATA COLLECTION 11
E. PROCEDURES: MEASUREMENTS & RECORDINGS 12
1. Clinical assessment 12
(a) Disease severity measures 12
(b) Family history. 13
(c) Diagnostic Criteria 13
2. Cognitive-Neuropsychological assessment 13
3. SES-SDH 13
4. Phlebotomy and DNA 14
5. MRI recordings 14

Protocol Title: MULTI-PARTNER CONSORTIUM TO EXPAND DEMENTIA RESEARCH IN LATIN AMERICA (ReDLat)

Funding: NIH-NIA-R01 (US-South American initiative for genetic-neural-behavioral interactions in human neurodegenerative research) / Alzheimer's Association (AA) / Tau Consortium (TC) / Global Brain Health Institute (GBHI)

Version of Protocol/Date of Version: March 2020

Abbreviation List

AD: Alzheimer's disease
FTD-ALS: Amyotrophic Lateral Sclerosis
AO: age of onset
BHA: Brain Health Assessment
CBS: Cortico-Basal syndrome
FTDs: frontotemporal dementia spectrum
FTLD: Frontotemporal lobar degeneration
BvFTD: Behavioral variant frontotemporal dementia
GAD-7: Generalized Anxiety Disorder
GDS: Geriatric Syndromes Assessment
GWAS: Genome-wide association studies
HIC: High income countries
HSES: High socio-economic status
IFS: Ineco Frontal Screening
LSES: Low socio-economic status
MRI: Magnetic resonance imaging
NACC: National Alzheimer's Coordinating Center
nfvPPA: Nonfluent variant Primary Progressive Aphasia
NPI-Q: Neuropsychiatric Inventory Questionnaire
PCA: Principal Component Analysis
PD: Project Director
PI: Principal Investigator
PRS: Polygenic risk score
rs-fMR: Resting state-functional MRI
LAC: Latin American Countries
LAC-CD: Latin America and Caribbean consortium on Dementia
SDH: Social Determinants of Health
SES: Socioeconomic Status
SAC: South American Countries
svPPA: Semantic variant Primary Progressive Aphasia
UDS: Uniform Data Set
YaD: Years after diagnosis

A. PROTOCOL INFORMATION

Project Directors: Agustin Ibanez, Jennifer Yokoyama, Kate Possin, Francisco Lopera, Kenneth Kosik, Victor Valcour, Bruce Miller.

Principal Investigators: Jennifer Yokoyama, Katherine Possin, Ricardo Nitrini, Leonel Takada, Nilton Custodio, Diana Matallana, Francisco Lopera, Ana Luisa Sosa, Alberto Avila-Funes, Andrea Slachevsky, María Isabel Behrens, Juan Felipe Cardona.

Research Coordinators: Maira Okada de Oliveira, Stefanie Piña Escudero, Maritza Pintado

Program Managers: Shireen Javandel (UCSF), M. Eugenia Godoy (Argentina), Ines Menvielle and Gonzalo Roca (administrative team)

Site Investigators

Argentina
PI: Agustin Ibanez (agustin.ibanez@gbhi.org)
Coordinator: Adolfo Garcia (adolfomartingarcia@gmail.com)
Program Manager: Maria Eugenia Godoy (mariaeugeniagodoyr@gmail.com)
Administrative team: Ines Menvielle inemenvielle@gmaill.com), Gonzalo Roca (gonzaroca@gmail.com)
Other Co-Investigators: GARCIA; ODONELLY; PASCARIELLO; BAEZ; GONZALES CAMPO; BIRBA; FITTIPALDI; DOTTORI; SEDEÑO; GARCIA CORDERO; BRUNO; ROCA; PARRA; MOGUILNER; HESSE

Brazil
PI: Ricardo Nitrini (rnitrini@uol.com.br), Leonel Takada (leonel.takada@gmail.com)
Coordinator: Maira Okada de Oliveira (maira.okada-de-oliveira@gbhi.org),
Administrative team: Maira Okada de Oliveira, Leonel Takada
Other Co-Investigators: TAKADA; YASSUDA; DA COSTA LEITE; DOZZI BRUCKI, CARAMELLI, RESENDE

Colombia (Medellin)
PI: Francisco Lopera (floperar@gmail.com)
Coordinator: Natalia Trujillo (natalia.trujillo@udea.edu.co)
Administrative team: Sonia Perez (pfinanciera1@undacionudea.co), Angela Restrepo (dirjuridica@fundacionudea.com)
Other Co-Investigators: PINEDA; ZARATE MONTOYA; URIZA CARRASCO; BOCANEGRA; TRUJILLO

Colombia (Bogota)
Key personnel: Diana Matallana (dianamatrossi@gmail.com; dianamat@javeriana.edu.co)
Coordinator: Carlos Cano (ccano@javeriana.aedu.co)
Administrative team: Caludia Giselle Tovar (claudia.tovar@javeriana.edu.co); Johana Contreras (l.contreras@javeriana.edu.co), Jeny Severiche (jseveriche@javeriana.edu.co)
Other Co-Investigators: SANTAMARÍA; BAEZ, SUAREZ, REYES, SANTACRUZ, ZARANTE, GIRALDO.

Colombia (Cali)
Key personnel: Juan Felipe Cardona (felipe.cardona@correounivalle.edu.co)

Peru
PI: Nilton Custodio (niltoncustodio@neuroconsultas.com; ncustodio@ipn.pe)
Coordinator: Rosa Montesinos (rmontesinos@ipn.pe)
Administrative team: David Lira (dlira@ipn.pe)
Other Co-Investigators: PINTADO, CHAVEZ; MONTESINOS; CUENCA & CASTRO-SUAREZ

Mexico (Sosa)
PI: Ana Luisa Sosa Ortiz (drasosa@hotmail.com)
Coordinator: Mariana Longoria Ibarrola (mariana.longoria@gbhi.org)
Administrative team: Dr. Miguel Angel Celis López (direcciongeneral@innn.edu.mx)
Other Co-Investigators: Isaac Acosta,Rosa Elena Flores Montes, Rocío Ramírez Santos

Mexico (Avila-Funes)
PI: Alberto Avila-Funes (avilafunes@live.com.mx)
Coordinator: Stefanie Danielle Piña Escudero (stefanie.pina-escudero@gbhi.org)
Administrative team: Rosa María Hernández (coordinacion@funsaed.com)
Other Co-Investigators: AGUILAR NAVARRO, MIMENZA ALVARADO, MUTCHINICK BARINGOLTZ, SAMUDIO CRUZ, GRANADOS DOMINGUEZ

Chile (Slachevsky)
PI: Andrea Slachevsky (andrea.slachevsky@uchile.cl)
Coordinator: Loreto Castro (castro@hsalvador.cl)
Administrative team: Daniel Berger (dbergez@uchile.cl)
Other Co-Investigators: SAN MARTIN, ROGERS, MORE, DURAND, SANTIBAÑEZ, PARRAO, ARANGUIZ, SUNKEL, LILLO, DELGADO, GOMEZ, LEON, OLAVARRIA, GONZALEZ, HENRIQUEZ

Chile (Behrens)
PI: Maria Isabel Behrens (behrensl@uchile.cl)
Coordinator: Daniela Ponce (dponcedelavega@gmail.com)
Administrative team: Patricia Ayala (patyeliz@gmail.com)
Other Co-Investigators: Included in Slachevsky team.

Chile (CSCN)
PI: Agustin Ibanez (agustin.ibanez@gbhi.org)
Coordinator: David Huepe (david.huepe@gmail.com)
Administrative team: Nicolas Rojas Osorio (rojas.o.nicolas@gmail.com)
Other Co-Investigators: CROSSLEY, OSSANDON

US
PI: Jennifer Yokoyama (Jennifer.Yokoyama@ucsf.edu), Kate Possin (kate.possin@gbhi.org), Victor Valcour (Victor.Valcour@ucsf.edu), Kenneth Kosik (kosik@lifesci.ucsb.edu), Bruce Miller (Bruce.Miller@ucsf.edu; bruce.miller@gbhi.org)
Coordinator: Jennifer Yokoyama (Jennifer.Yokoyama@ucsf.edu)
Program Manager: Shireen Javandel (Shireen.Javandel@ucsf.edu)
Administrative team: Elena Wright (Elena.Wright@ucsf.edu), Vera Bakman (Vera.Bakman@ucsf.edu)
Other Co-Investigators: SEELEY; ROSEN; RANKIN, GEIER, & KRAMER

Associate researchers, key personnel, OSC, and team structure:

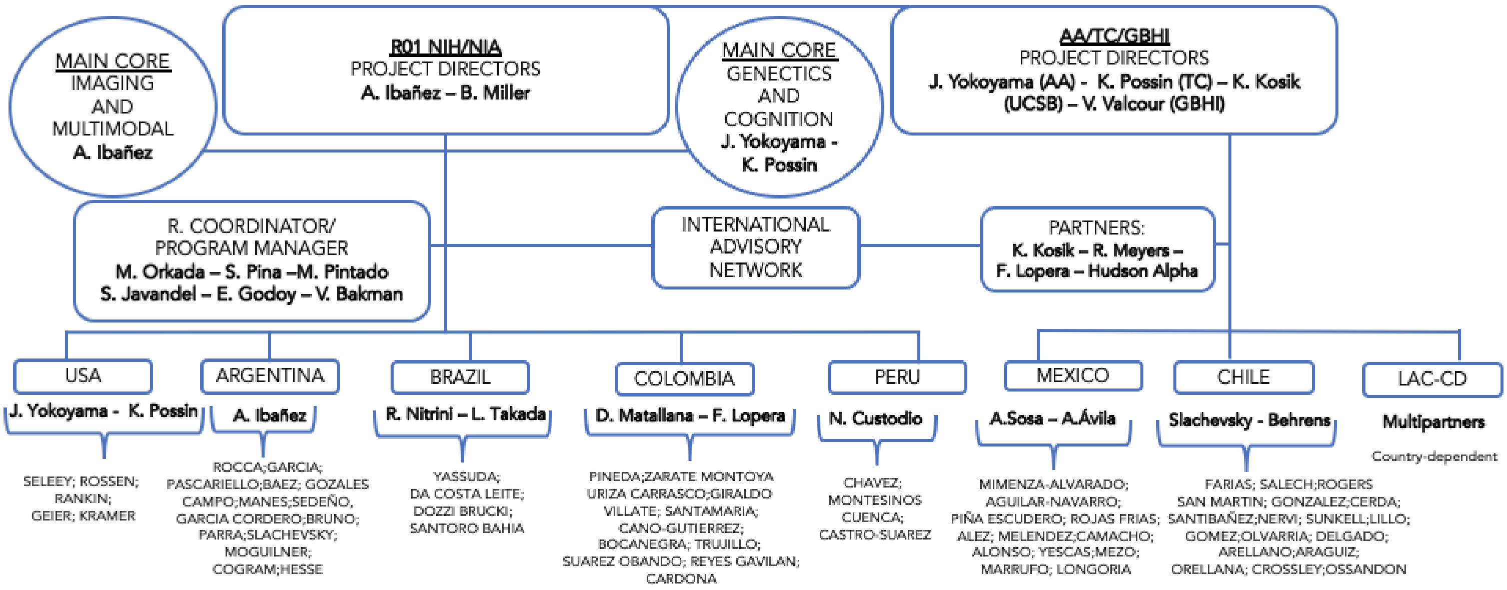

Institutions and Reviewing IRB's:

1. Argentina: INECO-Centro de Psicología Médica San Martín de Tours: FWA00028264
2. Brazil: Hospital das Clínicas da Faculdade de Medicina da Universidade de Sao Paulo: FWA00001035
3. Colombia: Universidad de Antioquia-SIU: FWA00028864
4. Colombia: Pontificia Universidad Javeriana - Hospital Universitario San Ignacio: FWA00001113
5. Peru: Hospital Nacional Docente Madre Niño San Bartolome FWA00010121
6. Mexico: Instituto Nacional de Ciencias Médicas FWAA00014416
7. Mexico: Instituto Nacional de Neurologia: FWA00008475
6. Chile: Geroscience FONDAP. Universidad de Chile (TBD)
7. Chile: Hospital Clinico, Universidad de Chile: FWA00029089
8. Chile: Universidad Adolfo Ibáñez (TBD)
9. USA: UCSF-MAC: FWA00000068

Source of Funding: NIH/NIA 1R01AG057234 - 01A1, Alzheimer's Association (AA) / Tau Consortium (TC)/ Global Brain Health Institute (GBHI)

Study Duration: This is a cross-sectional study with no planned follow-up of participants; although all will consent to be contacted for follow-up should funding be identified. The total duration of the recruitment period is five years.

Anticipated Start Date: 09/30/2019 (initial) 04/01/2020 (recruitment)

Anticipated Completion Date: 03/31/2024 (+ 6/12 month extension)

Required Number and Type of Study Subjects: up to 4,200 participants, including patients diagnosed with Alzheimer's disease (AD) and frontotemporal dementia spectrum (FTDs) disorders (e.g. behavioral variant FTD, progressive supranuclear palsy, corticobasal degeneration and non-logopenic Primary Progressive Aphasia, Amyotrophic Lateral Sclerosis), together with a demographically matched group of healthy controls, female and male, aged 40-80 years. Also families with familial presentations of AD/FTD will be included.

B. STUDY SUMMARY

General Aim and hypothesis

To identify the unique genetic and social determinants of health/socio-economic status (SDH/SES) factors that drive AD and FTDs presentation in Latin American Countries (LAC) relative to the US, including risk factors, cognitive profiles, and brain imaging. We will establish a first-in-class cohort anchored in at least six LAC (currently: Argentina, Chile, Colombia, Brazil, Mexico, and Peru), compared to US samples (totaling > 4200 participants, including 2100 controls, 1050 AD participants, and 1050 FTDs participants). We will also assess novel families across LAC via the Latin America and Caribbean consortium on Dementia (LAC-CD). In addition to the strategy based on patients with sporadic presentations tested for genetic risks (risk scores), the project will also support recruitment of AD and FTD families with an autosomal dominant-like presentation from the LAC-CD. We will couple standardized clinical assessments with innovative analytical techniques to account for heterogeneity in these diverse populations (Fig 1).

General hypothesis: There are unique risk factors for AD and FTDs in LAC (e.g., genetic risk factors enriched in LAC populations; underlying cognitive and neural vulnerability due to SES/SDH) compared to US populations. Our plan to recruit large numbers of controls and patients across these diverse populations will provide opportunities to identify new genetic and SES/SDH risks for AD and FTD. In addition, the machine learning strategies will reduce the impact of heterogeneity and will allow us to refine the accuracy of association studies.

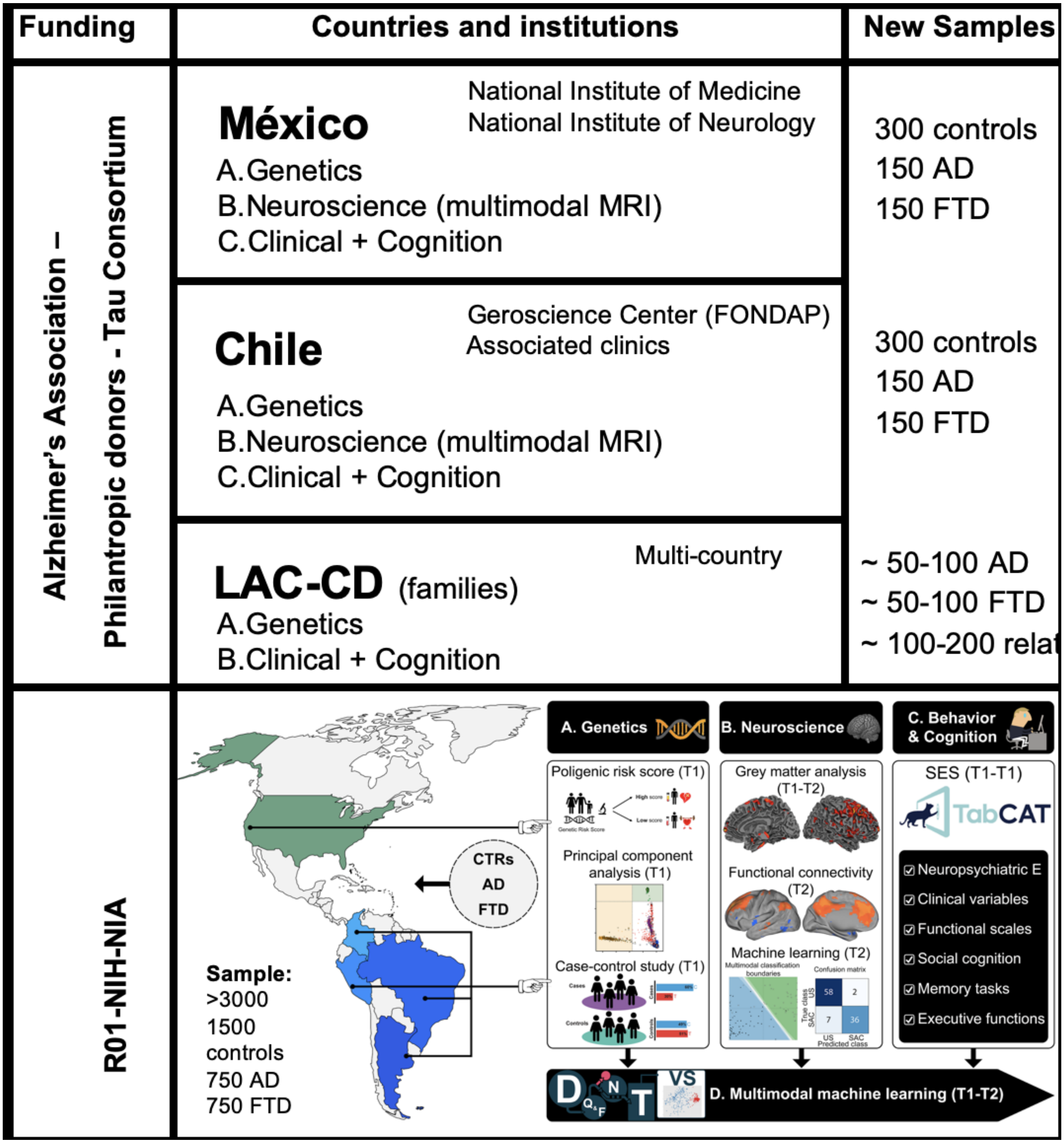

Figure 1. Long-term goals and proposal. Systematic comparisons between LAC and US samples of AD and FTD via a novel, multimodal approach. The top panels show the extended proposal to Mexico, Chile, and LAC-CD. The bottom panel shows the R01 proposal. The multimodal patterns will be assessed with different measures of (A) genetic risk (Aim 1), (B) imaging markers boosted by computational approaches, and (C) harmonized and novel measures of cognitive profiles and SES/SDH (Aim 2). These data sources will be (D) integrated and compared among countries through machine learning (Aim 3) to unveil the main commonalities and differences between US and LAC samples. Tier 1 (T1): Larger study (Aim 1 & 3). Tier 2 - T2, smaller study with deep neurocognitive investigation (Aim 2 & 3). D: Data; Q&F: Quality & feature extraction; N: normalization; T: test; VS: visualization.

Specific Aims and Hypothesis

<u>Aim 1</u>: **To establish genetic contributions to AD and FTDs in diverse LAC cohorts (Tier 1 study, with a larger sample size than Tier 2).** By elucidating the genetic substructure and familial contributions to AD and FTDs in LAC relative to the US, we will be able to identify proper populations for replication of our genetic findings. By assembling this large cohort, we will also be well-positioned to establish a LAC-specific polygenic risk score (PRS) for predicting AD and FTDs risk in future samples.

a. **To identify the prevalence of autosomal dominant and rare variant risk factors for AD and FTDs in target genes**. **<u>H1A</u>:** *We hypothesize that, relative to the US, LACs have a higher frequency of familial forms of AD and FTDs. We anticipate that the discovery of new families with multiple affected individuals will advance efforts to treat AD and FTD in patients with rare mutations (PS1, APP, MAPT, GRN, and C9orf72).*

b. **To perform exploratory genome-wide association studies (GWAS) in LAC subpopulations**. **<u>H1B</u>:** We hypothesize that, relative to US participants, LAC are enriched for novel risk in genes that code for lipid metabolism (AD) and lysosomal function (FTD).

c. **To test whether polygenic risk scores (PRS) developed in European AD and FTDs populations prove valid in LAC**. **<u>H1C</u>:** We hypothesize that PRS will work best at discriminating participants with disease from controls in the European predominant subpopulation (US and, to a lesser extent, Argentina, Chile) than in the African and Indigenous-majority admixed cohorts (Peru, Brazil, Colombia, Mexico).

<u>Aim 2</u>: **To elucidate the impact of SES/SDH on clinical, cognitive, and brain imaging signatures in LAC and the US**. (Tier 2 study-comprehensive imaging and cognitive evaluation in a subset of Tier 1). To compare participants across regions, we need to establish standardized cognitive measures and understand how SES/SDH impacts the manifestations of dementia in LAC.

a. **To evaluate how SES moderates the relationship between age at onset and disease severity in AD and FTDs**. **<u>H2A</u>:** We hypothesize that AD and FTDs will emerge at an earlier age in patients with low-SES vs. high-SES (dichotomized), and measures of disease severity, including cognitive performance, and multimodal neuroimaging, will be worse in the low-SES group even after accounting for age.

b. **To assess the differential impact of SES/SDH on clinical, cognitive and brain imaging deficits in LAC vs. US samples**. **<u>H2B</u>:** We hypothesize that differences in disease severity ratings, cognition, and multimodal neuroimaging that reflect low vs. high SES disparities will be greater in LAC patients compared to US patients.

<u>Aim 3</u>: **To determine whether genetic risk and SES/SDH yield better discrimination between LAC and US patients as compared with other cognitive, neuroimaging, and clinical variables** (Tier 1 & 2). To date, no study has sought to establish which potential predictors prove more sensitive to discriminate between LAC and US patients. In particular, although genetic risk and SES/SDH (Aims 1 and 2) have the potential to robustly differentiate between such samples, no study has explored their role, let alone as compared to other multimodal factors. To address this issue, we will apply data-driven machine-learning analysis to determine the top factors that best discriminate patients in LAC from those in the US. Multimodal measures from controls of each country will be used for population-specific normalization of patient data. **<u>H3</u>:** It is hypothesized that the main features that better discriminates between LAC and US patients, will be related to SES/SDH and genetic risk (e.g. standardized PRS) compared to other variables (clinical, cognitive and imaging measures).

C. SUBJECTS AND INCLUSION/EXCLUSION CRITERIA

<u>Participant Selection</u>: Study participants and controls will be recruited from each center in each country (USA: UCSF Memory and Aging Center, MAC; Argentina: INECO-INCYT; Brazil: Universidade de São Paulo; Colombia: Universidad de Antioquia and Universidad Javeriana; Peru: Peruvian Institute of

Neuroscience; Mexico: Instituto Nacional de Ciencias Médicas and Instituto Nacional de Neurologia; Chile: Fondap Center, Hospital Clinico at the Universidad de Chile, and Universidad Adolfo Ibáñez). All participants will receive a clinical, neurological, and neuropsychological evaluation, as previously reported for FTD and AD. All participants will undergo a similar multi-step screening process with in-person visits at each clinical center, including a neurologic exam, blood extraction and storage, cognitive assessment, familial antecedent survey, medical history, and imaging session. An informant will be present in all cases. All participants will provide written informed consent in agreement with the Helsinki declaration, NIH guidelines, and local regulatory boards.

Although all protocols (cognitive assessments, phlebotomy, and imaging session) will be routinely performed in all AD patients, FTD patients, relatives, and controls in all involved countries, we do anticipate a modest missing data rate (~-5-10% for cognition/phlebotomy and ~20% for imaging). We have attempted to account for this expected amount of missing data by recruiting slightly more than the goal total number of samples per site. We do not plan to impute data that is missing but will instead modify each analysis based on the amount of data available. In the case we have more than 30% of missing data, we will include re-assessment and/or new recruitments to achieve the stated sample size goals.

Participants with Dementia

Inclusion Criteria:

1. Clinical diagnosis of mild/moderate AD or FTDs (including bvFTD, nfvPPA, svPPA, fvPPA FTD-ALS, PSP, or CBS) using standard of care approaches by site principal investigator (but always applying current criteria for AD and FTD variants). Patients may show criteria-related atrophy (routine MRI) or hypoperfusion/hypometabolism (SPECT or PET) when available.
2. Age between 40 - 80 years
3. Minimum fluency (as required for assessment and judged by the clinician or evaluator) in English, Spanish or Portuguese depending on the language used for assessment
4. Visual and auditory ability adequate for cognitive testing as judged by the site investigators
5. Availability of a study partner (informant) who has frequent contact with the participant (e.g., family member, significant other, friend, caregiver) who is generally aware of the participants' daily activities and can provide information about the participant's cognitive and functional performance
6. Capable of providing informed consent or be accompanied by a legally authorized representative or guardian to do so.

Exclusion Criteria:

1. CDR=3 (for AD) or FTLD-CDR (FTD) =3. This criteria does not apply for phlebotomy in the case of relatives or patients from familial presentations.
2. Intoxication at the time of evaluation, multiple systems atrophy, brain tumor, multiple sclerosis, prion disease, Huntington's disease, intracerebral hemorrhage
3. Only for Tier 2: Presence of ferromagnetic implants that impact MRI acquisition
4. When routine MRI is available: Clinically significant cerebrovascular disease, mass lesion revealed on MRI (when available), which in the opinion of the attending neurologist could affect cognition and behavior or confound imaging analysis.
5. B12 deficiency (B12 < normal), hypothyroidism (TSH >150% of normal), HIV infection, renal failure (creatinine > 2), liver failure (AST > 2x normal), respiratory failure (requiring oxygen), dementia due to other than FTLD, PSP, CBD or AD, extra-axial brain tumor (with visible compression of the brain parenchyma), cerebral infarct, large confluent white matter lesions (grade 3 on the Fasekas scale), other significant major systemic disease (as judged by the attending neurologist).
6. Core clinical criteria for other dementia types or other neurologic disorders.
7. Unable to read and write in English, Spanish or Portuguese depending on the language used for assessment

Healthy Control Participants

Inclusion Criteria:

1. Healthy, community dwelling individuals with CDR of 0
2. Age between 40 - 80 years
3. For standardization, normalization and machine learning's probability calculations, controls will be similar in age-, sex-, SES-, and education with patients. For data recruitment we will use the following algorithm: a) select controls whiting the anticipated broad range; b) once we have a minimum of patients and controls, will use a MATLAB based script algorithm to highlight the control profiles requested to keep the <2-3 SD of each matching; c) perform specific search for controls with the required parameters.
4. Minimum fluency in English, Spanish or Portuguese depending on the language used for assessment
5. Visual and auditory ability adequate for cognitive testing as judged by the site investigator
6. Capable of providing informed consent or be accompanied by a legally authorized representative or guardian to do so.
7. A reliable informant who has frequent contact with the participant and is available to provide information about the participant is required.

Exclusion Criteria:

1. Known diagnosis of mayor cognitive, neurological, or psychiatric illness
2. History of alcohol or other substance abuse
3. Family history of AD or FTDs (first grade)
4. For Tier 2 only: Presence of ferromagnetic implants that impact MRI acquisition
5. Unable to read and write in English, Spanish or Portuguese depending on the language used for assessment

Relatives

Participants include relatives coming from AD and FTD families with an autosomal dominant-like presentation from the **LAC-CD.** The minimum protocol assessment for these participants includes a phlebotomy and a clinical evaluation.

Inclusion Criteria:

Participants should be 18 years of age or above and have familial aggregation (minimum three relatives with dementia in at least two generations). Potential younger participants will be discussed by the local PI and PDs. A reliable informant who has frequent contact with the participant and is available to provide information about the participant is required. Known diagnosis of major cognitive, neurological, or psychiatric illness will not be an exclusion criteria.

Exclusion criteria:

Inability to communicate in English, Spanish or Portuguese depending on the language used for assessment.

D. STUDY DATA COLLECTION

Tier 1: We will assess 4200 participants. We will target 300 controls, 150 AD and 150 FTDs per country in the initial 7 countries (10 sites). We will also assess (phlebotomy and clinical evaluation) novel families across LAC via the Latin America and Caribbean consortium on Dementia (LAC-CD). Enrollment progress will be monitored and enrollment targets per country will be modified based on site capacity and performance to achieve our goals.

Tier 2: From the Tier 1 study, we will select a subset of 1680 participants (120 controls, 60 AD and 60 FTD patients per country, totaling 840 controls, 420 AD, 420 FTD patients) for further in-depth evaluation (additional cognitive and MRI assessments). In this Tier 2 study, to accomplish Aim 2, a subset of participants will be included when they present with high SES (HSES) or low SES (HSES). *Grouping variables:* To classify subjects as HSES vs LSES, we will use values above and below median SES scores (divisor value) as a grouping factor. These values will be calculated at the end of year 1, and the more extreme values (not representing more than 35% of the sample) evaluated each year will be selected after control for age and sex. The same procedure will be implemented the following years. Also, and beyond this criteria, other participants may be included in Tier 2 when relevant for research purposes.

Data collection (Table 1) will be organized in four stages (the first two for Tiers 1 and 2, and the other two for Tier 2 only). **First**, a clinical diagnosis will be established through a local standard examination –including extensive neurological, neuropsychiatric, and neuropsychological assessments– and case revision by the local multidisciplinary clinical experts. At this time, the team should record the clinical impression and apply existing diagnostic criteria. Consent, measures of severity, as well as checking for inclusion/exclusion criteria will be performed at this stage when possible, the UDS cognitive assessment will be performed in this stage also). **Second**, a 2-3 hour neuropsychological evaluation will be performed, including standardized questionnaires and cognitive tests. Self-report questionnaires and SES/SDH evaluations for controls and relatives of the patients will be sent for completion via interview, phone, email, or online (i.e., REDCap) during this stage. Also, during this stage, a blood extraction protocol will be implemented by participating researchers or trained hospital staff at the clinical and hospital centers associated with this project. **Third**, for the behavioral assessment in Tier 2, we will incorporate pending assessments from the protocol to complete cognitive tasks. **Fourth**, a one-hour session will be performed for imaging recordings (see section E, procedures), including structural, FLAIR, and resting state-functional MRI (none of these procedures requires the use of contrast). Each participant will be followed up and monitored during each stage to ensure that the whole evaluation process is completed, thus minimizing data loss. Also, when appropriate, they will receive payment to cover transportation costs or other costs related to the loss of hours of their working day.

Research material obtained from the subjects includes demographic information, SES/SDH, neuropsychological data, functional status evaluations, neuroimaging data, and biological specimens (blood and DNA). No personal identifiers will be labeled on specimens or databases. Existing medical records, including records from other physicians and MRI, may also be accessed during all phases of the project. To protect personal data, all participant data will be coded in a confidential manner using anonymous identification numbers to protect the identity of the participant (PIDN, Participant Identification Number), and all data entered into the password-protected private database will only utilize this identifier. A list of subject names and associated codes will be kept in a separate, secure location. Electronic records will be stored in a secure, password-protected network. All paper records will be kept in a locked file cabinet inside a locked office.

Table 1: Schedule of procedures for patients and control

Procedures	Clinical Screening # (Day -30 to -1)	Tier 1	Tier 2
Consent	X (30-40min)		
Contact information Form	X (5min)		
Inclusion/Exclusion criteria checklist	X (10-15min)		
Severity measures (AO, YaD, FTLD-CDR*, MMSE)	X (40min)		
Other clinical measures (GDS, GAD-7, NPI-Q*, Loneliness scale, PFAQ*)	X (30min)		
UDS Cognitive Assessment	X (30min)		
Diagnostic Criteria	X (10min)		
Brain Health Assessment (TabCAT)		X (10min)	
STMB + mini-SEA		X (20-30min)	
Family Tree* (Progeny)		X (30min)	
ReDLat socioeconomic status and social determinants of health questionnaire*		X (20 min)	
Spontaneous speech recording		X (5min)	
Phlebotomy (30ml) 3 tubes		X (20min)	
EXAMINER (TabCAT)			X (16-20min)
Rapid Naming Task			X (2 min)
MRI/FLAIR/rs-fMRI			X (45min)
**Total time**	**Approx. 3 hrs**	**Approx. 2 hrs**	**Approx. 1.5 hrs**

* to be completed by control participants and informant for cases
# Most of these evaluations will be part of the routine clinical assessment of each center

E. PROCEDURES: MEASUREMENTS & RECORDINGS

1. Clinical assessment

Participants will undergo a local defined complete clinical evaluation to examine cognitive symptoms, behavioral changes, psychiatric manifestations, and motor features, as reported in prior work.

(a) Disease severity measures

Disease severity measures will include informant ratings, condition-normalized age of onset (AO), and progression pattern (severity measures weighed by years after diagnosis, YaD). We will also include standard severity evaluations, namely: the CDR for AD, and frontotemporal lobar degeneration-CDR (FTLD-CDR) for FTD. We will also include measures of depression (GDS), anxiety (GAD-7) and personality (NPI-Q).

(b) Family history.

Family history interview will be performed through interview with the participant and informant. Data will be recorded using Progeny.

(c) Diagnostic Criteria

A standardized diagnostic assessment will be completed for each participant. This brief instrument (no more than 10 minutes to complete) will be filled out by the physician who examined the participant, with input from the neuropsychologist when possible. In addition to documenting the physician's clinical best estimate, formal criteria for AD and FTLD will be applied. The instrument may be completed at the time of referral or following a formal case review, and will be based on the clinical evaluation and performance on a comprehensive neuropsychological assessment, when available. For cases in which the comprehensive neuropsychological assessment has not been collected at the time of diagnosis, the instrument may be revised following that assessment.

2. Cognitive-Neuropsychological assessment

(a) Harmonized protocol

We will use the National Alzheimer's Coordinating Centers (NACC) neuropsychological battery of the Uniform Data Set (UDS, NB 3.0). It covers episodic memory, processing speed, executive function, language, constructional ability, and behavioral symptoms. When possible, the current version of the NACC FTLD module will be included with its questionnaires for a more detailed account of social-emotional and behavioral changes, such as the Revised Self-Monitoring Scale (RSMS) and the Interpersonal Reactivity Index (IRI).

(b) Extended harmonized and novel protocol*:* Two assessment batteries on TabCAT will be included: the Brain Health Assessment (BHA) and the NIH-EXAMINER. The BHA is an efficient and comprehensive assessment optimized for sensitivity to all types of neurodegenerative diseases, not just AD. In addition to a global score, the BHA produces subscores of memory, visuospatial, language, and executive functions that are important for characterizing neurocognitive profiles. The NIH EXAMINER will provide a detailed evaluation of executive functions that have been shown previously to differentiate AD from bvFTD and includes composite measures of set-shifting and inhibition, fluency, and working memory. Spontaneous speech recording for automated analysis will be also included.

(c) Disease sensitive tasks (STMB & Social cognition): We will use the short version of the STMB task developed by Mario Parra (7-10 min). For social cognition in FTD, we will combine short tasks by using the mini-SEA, providing scores of emotional recognition and theory of mind. Both combined tasks require a total time of 20-30min.

(d) LAC local protocol: As recommended, we will include local instruments for dementia characterization as an additional control (via covariation or PCA assessment, see below). This evaluation is not time-consuming as these tasks are routinely applied in each local center, independently of this protocol.

3. SES-SDH

SES: Global measures of SES and Social Determinants of Health (SDH) will be assessed with a UCSF-ReDLat questionnaire that includes previously validated dimensions.

4. Phlebotomy and DNA

Blood samples will be collected in in EDTA precoated tubes.The blood (BD Hemogard™ tubes) will be separated into plasma and cellular fractions by centrifugation at 1500xg for 15 min. Genomic DNA will be extracted from leukocytes using a Genomic DNA extraction kit (Promega) following the manufacturer's instructions.

5. MRI recordings

Multicenter recordings will be obtained in 3T scanners included in the project (Argentina: Siemens Magnetom Multitransmit; Brazil: Philips Achieva; Chile: Philips Ingenia; Colombia: Philips Achieva; Mexico: Philips Achieva; Peru: Siemens Magnetom Verio; US: Siemens Prisma Fit).

(a) T1: Whole-brain structural T1-rapid gradient-echo scans will be acquired

(b) rs-fMRI: Ten minute resting-state fMRI scan (instruction: “Do not think about anything in particular”)

(c) Vascular control: Finally T2* and FLAIR sequences will assess vascular effects.
