# Supplementary material for: The Multi-Partner Consortium to Expand Dementia Research in Latin America (ReDLat): Driving Multicentric Research and Implementation Science
Source: Front Neurol. 2021 Mar 11;12:631722. doi: 10.3389/fneur.2021.631722 (PMC7992978; doi:10.3389/fneur.2021.631722)
Supplement: Supplementary file 2 [file Data_Sheet_2.PDF]

Diagnostic Evaluation

PIDN: __ __ __ __ __   Date: __ __ / __ __ / __ __ __ __   Physician Completing Form: _______________

Essential Components:

1. Must be fully completed for every participant enrolled
2. Must be completed by, or in close consultation with, the physician who evaluated the participant **or** the physician who supervised the clinical evaluation
3. Must be completed or revised following comprehensive neuropsychological assessment

1. Diagnosis Method – responses in this form are based on diagnosis by:

   □1 A single clinician □2 A formal consensus panel □3 Other (e.g., two or more clinicians or other informal group)

2. Has a comprehensive neuropsychological evaluation been completed at this time?

   □0 No

   □1 Yes

3. Are you using imaging to complete this form?

   □0 No

   □1 Yes

4. Does the participant have normal cognition, behavior, and function? (If CDR is available it should be 0, if neuropsychological testing is available it should be within normal range.)

   □0 No (CONTINUE TO QUESTION 5)

   □1 Yes (SKIP TO ETIOLOGIC DIAGNOSES)

5. Does the participant meet criteria for dementia?

   ALL CAUSE DEMENTIA CRITERIA

   The participant has cognitive or behavioral symptoms that meet all of the following criteria:

   - *Interfere with ability to function as before at work or at usual activities*
   - *Represent a decline from previous level of functioning*
   - *Are not explained by delirium or other major psychiatric disorder*
   - *Include cognitive impairment detected and diagnosed through a combination of 1) history taking and 2) objective cognitive assessment*

   AND impairment in one or more of the following domains:

   - *Impaired ability to acquire and remember new information*
   - *Impaired reasoning and handling of complex tasks, poor judgment*
   - *Impaired visuospatial abilities*
   - *Impaired language functions*
   - *Changed in personality, behavior, or comportment*

   □0 No (SKIP TO QUESTION 7)

   □1 Yes (CONTINUE TO QUESTION 6)

6. If the participant meets criteria for dementia, select which domains are affected and identify the first and second (optional) symptom domain *and then* CONTINUE TO ETIOLOGIC DIAGNOSES

**Affected domains**	**No**	**Yes**	**First Symptom *(select one)***	**Second Symptom *(optional)***
6a. Memory	□0	□1	6a1. □1	6a2. □1
6b. Language	□0	□1	6b1. □1	6b2. □1
6c. Attention	□0	□1	6c1. □1	6c2. □1
6d. Executive	□0	□1	6d1. □1	6d2. □1
6e. Visuospatial	□0	□1	6e1. □1	6e2. □1
6f. Behavior	□0	□1	6f1. □1	6f2. □1
6g. Motor	□0	□1	6g1. □1	6g2. □1
6h. Constitutional	□0	□1	6h1. □1	6h2. □1

7. If the participant does not have normal cognition or behavior and is not clinically demented, indicate the type of cognitive impairment below

MCI CLINICAL CRITERIA

The participant has cognitive or behavioral symptoms that meet all of the following criteria:

- *The participant, informant, or clinician is concerned about a change in cognition compared to the participant's previous level*
- *There is impairment in one or more cognitive domains (memory, language, executive function, attention, visuospatial skill, behavior) based on cognitive testing performance or the clinician's judgment*
- *There is largely preserved independence in functional abilities (no change from prior manner of functioning or using minimal aids or assistance)*

Select one syndrome from 7a -7e as being present and then CONTINUE TO ETIOLOGIC DIAGNOSES

**Type**	**Present**	**Affected domains**	**No**	**Yes**
7a. Amnestic MCI, single domain	□1			
7b. Amnestic MCI, multiple domains	□1	Check yes for at least one additional domain (besides memory):		
		7b1. Language	□0	□1
		7b2. Attention	□0	□1
		7b3. Executive	□0	□1
		7b4. Visuospatial	□0	□1
		7b5. Behavior	□0	□1
7c. Non-Amnestic MCI, single domain	□1	Check yes to indicate the affected domain:		
		7c1. Language	□0	□1
		7c2. Attention	□0	□1
		7c3. Executive	□0	□1
		7c4. Visuospatial	□0	□1
		7b5. Behavior	□0	□1
7d. Non-Amnestic MCI, multiple domains	□1	Check yes for at least two domains:		
		7c1. Language	□0	□1
		7c2. Attention	□0	□1
		7c3. Executive	□0	□1
		7c4. Visuospatial	□0	□1
		7b5. Behavior	□0	□1
7e. Cognitively impaired, not MCI	□1			

ETIOLOGIC DIAGNOSES

This section must be completed for all participants. Indicate presumptive etiologic diagnoses of the cognitive disorder and whether a given diagnosis is a primary, contributing or non-contributing cause of the observed impairment, based on the clinician's best judgment. Only one diagnosis should be selected as primary.

<u>For participants with normal cognition</u>: Indicate the presence of any diagnoses by marking present, and leave the questions on whether the diagnosis was primary, contributing, or non-contributing blank.

**Etiologic diagnoses**	**Present**	**Primary***	**Contributing**	**Non-contributing**
8. Alzheimer's disease	□1	8a. □1	□2	□3
9. Frontotemporal lobar degeneration				
9a. Progressive supranuclear palsy	□1	9a1. □1	□2	□3
9b. Corticobasal degeneration	□1	9b1. □1	□2	□3
9c. FTLD with motor neuron disease	□1	9c1. □1	□2	□3
9d. FTLD not otherwise specified	□1	9d1. □1	□2	□3
10. Parkinson's disease	□1	10a. □1	□2	□3
11. Vascular brain injury<br>11b. Previous symptomatic stroke?<br>□0 No (SKIP TO QUESTION 11c)<br>□1 Yes<br>11b1. Temporal relationship between stroke and cognitive decline?<br>□0 No<br>□1 Yes<br>11b2. Confirmation of stroke by neuroimaging?<br>□0 No<br>□1 Yes<br>□9 Unknown<br>11c. Imaging evidence of cystic infarction in cognitive network(s)?<br>□0 No<br>□1 Yes<br>□9 Unknown<br>11d. Imaging evidence of cystic infarction, imaging evidence of extensive white matter hyperintensity (CHS grade 7-8+, *see photo*), and impairment in executive function?<br>□0 No<br>□1 Yes<br>□9 Unknown	□1	11a. □1	□2	□3

11e. Cortical cognitive deficit (e.g., aphasia, apraxia, neglect)
□0 No
□1 Yes
□8 Not Assessed

11f. Focal or other neurological findings consistent with SIVD (subcortical ischemic vascular dementia)
□0 No
□1 Yes
□8 Not Assessed

	**Left**			**Right**		
	**Yes**	**No**	**Not assessed**	**Yes**	**No**	**Not assessed**
11g. Motor (may include weakness of combinations of face, arm, and leg; reflex changes; etc.)	□1	□0	□8	□1	□0	□8
11h. Cortical visual field loss	□1	□0	□8	□1	□0	□8
11i. Somatosensory loss	□1	□0	□8	□1	□0	□8

12. Essential tremor	□1	12a. □1	□2	□3
13. Traumatic brain injury<br>13b. If present, are there symptoms consistent with chronic traumatic encephalopathy?<br>□0 No<br>□1 Yes<br>□9 Unknown<br>13c. TBI with brief loss of consciousness (<5 minutes)<br>□0 No<br>□1 Single<br>□2 Repeated/multiple<br>□9 Unknown<br>13d. TBI with extended loss of consciousness (≥5 minutes)<br>□0 No<br>□1 Single<br>□2 Repeated/multiple<br>□9 Unknown<br>13e. TBI without loss of consciousness (as might result from military detonations or sports injuries)<br>□0 No<br>□1 Single<br>□2 Repeated/multiple<br>□9 Unknown<br>13f. Year of most recent TBI: __ __ __ __	□1	13a. □1	□2	□3
14. Normal pressure hydrocephalus	□1	14a. □1	□2	□3
15. Epilepsy	□1	15a. □1	□2	□3
16. CNS neoplasm<br>16b. □1 Benign □2 Malignant	□1	16a. □1	□2	□3
17. Amyotrophic Lateral Sclerosis	□1	17a. □1	□2	□3
18. CNS Infection	□1	18a. □1	□2	□3
19. Autoimmune Disorder	□1	19a. □1	□2	□3
20. Active depression<br>20b. If present, select one:<br>□0 Untreated<br>□1 Treated with medication or counseling	□1	20a. □1	□2	□3
21. Bipolar disorder	□1	21a. □1	□2	□3
22. Schizophrenia or other psychosis	□1	22a. □1	□2	□3
23. Anxiety disorder	□1	23a. □1	□2	□3
24. Delirium	□1	24a. □1	□2	□3
25. Post-traumatic stress disorder	□1	25a. □1	□2	□3
26. Other psychiatric disease	□1	26a. □1	□2	□3
27. Cognitive impairment due to alcohol abuse	□1	27a. □1	□2	□3
28. Cognitive impairment due to other substance abuse	□1	28a. □1	□2	□3
29. Cognitive impairment due to systemic disease/ medical illness	□1	29a. □1	□2	□3
30. Cognitive impairment due to medications	□1	30a. □1	□2	□3

**For the primary diagnosis, physician will be asked to indicate the level of confidence in this diagnosis*

□1 Extremely Confident □2 Moderately Confident □3 Unsure of Some Elements □4 Not Confident

AD CRITERIA

This section must be completed for all participants who do not have normal cognition.

31. Has the onset of symptoms been insidious (i.e., gradual over the course of months or years, rather than over hours or days)?

□0 No

□1 Yes

32. Is there a clear-cut history of worsening cognition by report or observation?

□0 No

□1 Yes

33. In which of the following domains are the initial and most prominent cognitive deficits evident on history and examination

□1 Memory

□2 Language

□3 Visuospatial

□4 Executive

□5 Behavior

□6 Psychiatric

34. There is no evidence of a) substantial concomitant cerebrovascular disease, defined by a history of a stroke temporally related to the onset or worsening of cognitive impairment; or the presence of multiple or extensive infarcts or severe white matter hyperintensity burden; or (b) core features of Dementia with Lewy bodies other than dementia itself; or (c) prominent features of behavioral variant frontotemporal dementia; or (d) prominent features of semantic variant primary progressive aphasia or nonfluent/agrammatic variant primary progressive aphasia; or (e) evidence for another concurrent, active neurological disease, or a non-neurological medical comorbidity or use of medication that could have a substantial effect on cognition

□0 No

□1 Yes

FTLD CRITERIA

This section must be completed for all participants who do not have normal cognition.

35. Does the participant have an acquired and progressive difficulty with language?

□0 No (SKIP TO QUESTION 38)

□1 Yes

Are these features present on the current exam?	**Absent**	**Questionably present**	**Definitely present**	**Not evaluated**
35a. Poor object naming<br>(Core diagnostic feature of semantic variant; abnormal in all variants)	□0	□1	□2	□9
35b. Impoverished word selection/retrieval in spontaneous speech or writing<br>(Core diagnostic feature of logopenic variant; abnormal in all variants)	□0	□1	□2	□9
35c. Impaired word comprehension<br>(Core diagnostic feature of semantic variant; absent in other variants)	□0	□1	□2	□9
35d. Poor object/person knowledge<br>(Secondary or diagnostic feature of semantic variant; absent in other variants)	□0	□1	□2	□9
35e. Grammatical simplification or grammatical errors in speed or writing<br>(Core diagnostic feature of nonfluent/agramatic variant)	□0	□1	□2	□9
35f. Effortful, halting speech<br>(Core diagnostic feature of nonfluent/agramatic variant)	□0	□1	□2	□9

35g. Circumlocutory, empty speech<br>(Secondary diagnostic feature of logopenic variant; also present in semantic variant)	□0	□1	□2	□9
35h. Speech sound/word errors (paraphasias)<br>(Secondary diagnostic feature of logopenic variant; abnormal in nonfluent/agrammatic variant)	□0	□1	□2	□9
35i. Impaired speech repetition (inability to repeat verbatim sentence-length material)<br>(Core diagnostic feature of logopenic variant; present in nonfluent/agrammatic type; absent in semantic variant)	□0	□1	□2	□9
35j. Surface dyslexia and dysgraphia<br>(Secondary feature of semantic variant)	□0	□1	□2	□9

36. Is the participant's language syndrome consistent with PPA of a neurodegenerative type

AND

Is the language disorder the most prominent deficit at symptom outset and for the initial phase (1-2 years) of the disorder

□0 No (SKIP TO QUESTION 38)

□1 Yes (CONTINUE TO QUESTION 37)

37. Consensus diagnosis of dominant PPA subtype based on clinician and neuropsychologist judgment

□1 PPA, semantic variant

□2 PPA, nonfluent/agrammatic variant

□3 PPA, logopenic variant

□4 PPA, not otherwise specified

38. Are acquired alterations in behavior, personality, or comportment important elements in the clinical presentation of the subject?

□0 No

□1 Yes

Have the following symptoms/behaviors been prominent, persistent, and recurrent in (approximately) the past three years?	**Absent**	**Questionably present**	**Definitely present**	**Not evaluated**
38a. Disinhibition<br>Socially inappropriate behavior; loss of manners or decorum; impulsive, rash or careless actions	□0	□1	□2	□9
38b. Apathy or inertia<br>Loss of interest, drive, and motivation; decreased initiation of behavior	□0	□1	□2	□9
38c. Loss of sympathy/empathy<br>Diminished response to other people's needs or feelings; diminished social interest, interrelatedness, or personal warmth	□0	□1	□2	□9
38d. Ritualistic/ compulsive behavior<br>Simple repetitive movements or complex compulsive or ritualistic behaviors	□0	□1	□2	□9
38e. Hyperorality and appetite changes<br>Altered food preferences, binge eating, increased consumption of alcohol or cigarettes, oral exploration or consumption of inedible objects	□0	□1	□2	□9
38f. Changes on neuropsychological testing consistent with bvFTD<br>(refer to neuropsychological evaluation and neuropsychologist's impression)	□0	□1	□2	□9

38g. Impaired daily functioning<br>Are these alterations in behavior, personality or comportment the principal cause of impaired daily living activities?	□0	□1	□2	□9

39. Does the participant meet the criteria for clinical probable* or possible** bvFTD syndrome?

** PROBABLE: meets three of the above criteria in Questions 38a-f and (1) has impaired daily functioning;* ***and*** *(2) has imaging consistent with bvFTD*

** POSSIBLE: meets three of the above criteria in Questions 38a-f and either (1) is not functionally impaired;* ***or*** *(2) does not have imaging consistent with bvFTD*

□0 Does not meet criteria for bvFTD

□1 Probable bvFTD

□2 Meets criteria for possible bvFTD with impaired daily functioning but without evidence of diagnostic imaging

□4 Meets criteria for possible bvFTD but daily functioning is not significantly impaired

If participant has only one diagnosis (either PPA or bvFTD), then SKIP to QUESTION 41.

40. For participants with a diagnosis of both PPA and bvFTD, which diagnosis appeared first?

□1 bvFTD

□2 PPA, semantic variant

□3 PPA, nonfluent/agrammatic variant

□4 PPA, logopenic variant

□5 PPA, not otherwise specified

□9 Unknown

41. Please enter any additional notes critical for understanding the diagnosis.
